# Global Transcriptome Analysis of *Lactococcus garvieae* Strains in Response to Temperature 

**DOI:** 10.1371/journal.pone.0079692

**Published:** 2013-11-04

**Authors:** Mónica Aguado-Urda, Alicia Gibello, M. del Mar Blanco, José F. Fernández-Garayzábal, Victoria López-Alonso, Guillermo H. López-Campos

**Affiliations:** 1 Department of Animal Health, Faculty of Veterinary Sciences, Complutense University, Madrid, Spain; 2 Animal Health Surveillance Center (VISAVET), Complutense University, Madrid, Spain; 3 Bioinformatics and Public Health Department, Health Institute Carlos III, Madrid, Spain; 4 Health and Biomedical Informatics Research Unit, Medical School, University of Melbourne, Victoria, Australia; University Medical Center Utrecht, Netherlands

## Abstract

*Lactococcus garvieae* is an important fish and an opportunistic human pathogen. The genomic sequences of several *L. garvieae* strains have been recently published, opening the possibility of global studies on the biology of this pathogen. In this study, a whole genome DNA microarray of two strains of *L. garvieae* was designed and validated. This DNA microarray was used to investigate the effects of growth temperature (18°C and 37°C) on the transcriptome of two clinical strains of *L. garvieae* that were isolated from fish (Lg8831) and from a human case of septicemia (Lg21881). The transcriptome profiles evidenced a strain-specific response to temperature, which was more evident at 18°C. Among the most significant findings, Lg8831 was found to up-regulate at 18°C several genes encoding different cold-shock and cold-induced proteins involved in an efficient adaptive response of this strain to low-temperature conditions. Another relevant result was the description, for the first time, of respiratory metabolism in *L. garvieae*, whose gene expression regulation was temperature-dependent in Lg21881. This study provides new insights about how environmental factors such as temperature can affect *L. garvieae* gene expression. These data could improve our understanding of the regulatory networks and adaptive biology of this important pathogen.

## Introduction

*Lactococcus garvieae* is a ubiquitous and widely distributed microorganism that has relevance in veterinary and human medicine. Although this bacterium is one of the most important bacterial fish pathogens, affecting various wild and farmed fish species, particularly rainbow trout [1], it has also been isolated from other animal species, such as cows, buffalos, pigs, wild birds [2], cats, dogs, and horses [3]. *L. garvieae* has gained clinical relevance in human medicine during the last years, being considered an opportunistic and potentially zoonotic pathogen that causes a variety of infections [4]. In addition to its relevance as a pathogen, *L. garvieae* can also be isolated from rivers and sewage waters [2], and from different foods such as vegetables, meat and dairy products [5]. Recently, *L. garvieae* has also been isolated from fecal samples of healthy individuals, suggesting this microorganism could be either part of the human commensal microbiota or transient bacteria ingested with food [6]. This wide distribution of *L. garvieae* is likely related to its ability to adapt and survive in many environmental conditions including a wide range of pH (4.5 to 9.6), temperatures (from 10°C to 45°C), salinity concentrations (0 to 6.5%) and nutrient sources [7]. 

Bacteria usually respond to variations in environmental factors such as temperature with adaptive changes in their transcriptome [8-11]. Because *L. garvieae* is able to colonize multiple, diverse different environments, and because it causes infection in a broad range of different hosts, it must therefore be able to sense, adapt, and respond to these temperature fluctuations. Water temperature has been described as the most important environmental factor in the development of the *L. garvieae* infections in fish [1], but there is a complete lack of knowledge about the influence of temperature on *L. garvieae* gene expression. 

Over the last few years, functional genomics approaches, including transcriptomics, have been increasingly used to obtain global gene expression profiles, thereby providing a comprehensive view of microorganism physiology [12,13]. Although the genome sequences of several *L. garvieae* strains from different origins have been published recently [14-21], such global approaches have not yet been used to study the transcriptome of this pathogen. In the present study, we used microarray expression analysis to evaluate global transcriptional changes occurring in two clinical *L. garvieae* strains, isolated from fish and a human, respectively, when incubated at 18°C and 37°C. These temperatures correspond to that at which fish lactococcosis outbreaks usually occur and the physiological temperature in humans, respectively. This first transcriptome analysis of two *L. garvieae* strains demonstrated that this bacterium responds globally to temperature.

## Materials and Methods

### Bacterial strains and culture conditions

*Lactococus garvieae* strain 8831 (Lg8831) was isolated from diseased rainbow trout suffering lactococcosis [16], and *Lactococcus garvieae* strain 21881 (Lg21881) was isolated from a case of human septicemia [15]. The growth kinetics of Lg8831 and Lg21881 were studied at 18°C and 37°C. To minimize variation in experimental culture conditions and ensure reproducibility, a standardized inoculum was prepared by adding 2 mL of an overnight culture of Lg8831 or Lg21881 incubated at 29°C in 150 ml BHI broth. Lag-phase and bacterial growth rate were determined in three independent experiments at both temperatures by monitoring OD_600_ each hour until OD_600_ ~ 1.5 was reached. Differences in lag-phase and bacterial growth rate were assessed using the Fisher exact test with the software SPSS 19.0 (IBM, New York, USA). Differences were considered significant when p<0.05.

### RNA extraction and purification

Lg8831 and Lg21881 were grown aerobically in BHI broth (bioMérieux, Marcy l’Etoile, France) at several different temperatures (18°C, 29°C, and 37°C) and harvested at the mid-log phase (OD_600_ ~ 0.9) for RNA extraction. Total RNA from Lg8831 and Lg21881 was isolated from three independent samples (biological replicates) of each temperature condition by using RNeasy Protect Bacteria Mini Kit (Qiagen, Valencia, CA) according to the manufacturer’s protocol. Briefly, 1.5 mL of each culture sample were collected in 3 mL of RNAprotect and centrifuged. Pellets were resuspended in 200 µL of TE buffer containing 20 mg/mL of lysozyme (Sigma-Aldrich, Brondby, Denmark) and 20 µL of proteinase K solution (Qiagen), and incubated at RT for 60 minutes under agitation conditions. Total RNA extraction and purification was then performed using RNeasy kit columns including on-column DNA digestion. The quality and concentration of RNA was determined by using the RNA 6000 NanoKit on the Bioanalyzer 2100 (Agilent Technologies, Palo Alto, CA) at Genomics Unit facilities (Parque Científico de Madrid). Only high- quality RNA samples (RIN > 9) were used for the next step.

### Microarray and experimental design

A DNA microarray was designed based on the published Lg8831 [16] and Lg21881 [15] genome sequences. The microarray, containing 60 mer oligonucleotide specific probes for the 1992 and 2275 ORFs of the Lg8831 and Lg21881 genomes respectively, was designed using Agilent’s e-array software (https://earray.chem.agilent.com/earray/). The best probe method was used, and each probe was included in triplicate on the array. This custom oligonucleotide microarray was manufactured by Agilent Technologies on an 8x15K format. 

A dual-color reference design experiment was used [22]. Interrogated samples of Lg8831and Lg21881 were grown in triplicate at 18°C and 37°C, and RNA was extracted. These RNAs were independently labeled with Cy-5. A reference sample made of an equimolar mix of Cy-3-labeled mixture of Lg8831 and Lg21881 RNAs was used for all hybridizations. These reference RNAs were obtain from both strains grown separately at 29°C, quantified and then mixed for labeling. 

For the comparison of the Lg8831 and Lg21881 strain transcriptomic responses, a subset of common probes derived from those designed for Lg8831 was used. The criteria used for the selection of these subset of probes for the inter-strain comparisons was that probes designed for the strain Lg8831 should have a BLAT [23] similarity >98.4%, (no more than a single mismatch) with the Lg21881 genome. Thus, a total of 1302 probes (65% of probes for Lg8831) were selected and used.

### cDNA synthesis, labeling of total RNA, hybridization and scanning

A total of 5 µg of total RNA of each sample replicate was reverse transcripted and labeled by using the FairPlay III Labeling Kit (Agilent Technologies) according to the protocol provided by the manufacturer except for the following: purification of the dye-coupled cDNA was performed by using QIAquick PCR purification kit (Qiagen). The yield and the specific activity of each labeling reaction were determined in a NanoDrop 1000 spectrophotometer (NanoDrop Technologies, Inc., Rockland, DE). The hybridization and washing steps were carried out according to the Agilent’s “Two Color Microarray based Prokaryote Analysis” protocol. Image acquisition and scanning was performed on an Agilent G2565AA scanner according to the manufacturer’s instructions (GE2_107_Sep09).

### Data analysis

Image quantification was carried out using Agilent’s Feature Extraction Software v 8.5.1.1. Background-subtracted data were log-transformed, LOWESS-normalized, and analyzed for the detection of differentially expressed genes using BRB-ArrayTools Software developed by Dr. Richard Simon and the BRB-ArrayTools Development Team [24]. Triplicate probes on the microarray were averaged for the analyses. The SAM algorithm was used to identify the differentially expressed genes using a 0.05 proportion of false discoveries (FDR) with a 90% confidence. A further filtering step was carried out by selecting among SAM results only those whose expression ratio between the analyzed conditions was ≥2. 

Throughout this article we have used the term “up-regulated genes” to indicate those genes that were found differentially overexpressed at one temperature relative to the other.

### Microarray accession numbers

The data presented in this publication have been deposited in the Gene Expression Omnibus (GEO) database at the National Center for Biotechnology Information (NCBI) (http://www.ncbi.nlm.nih.gov/geo/) and are accessible through GEO Series accession number GSE40318.

### Validation of microarray data by reverse-transcription quantitative real-time PCR (RT-qPCR)

To validate the results obtained during the microarray analysis, RT-qPCR analysis was performed. A total of 10 genes were selected for the validation process. These genes were selected by taking the top three expressed and top two repressed genes at each comparison between the two temperatures for each strain. The *gyrA* gene was selected as an endogenous control because it did not present variation in its expression throughout the experiment. Triplicate assays utilizing the same three independent RNA samples used for the microarray hybridization were used for the RT-qPCR validation. The RT-qPCR primers were designed using Primer Express software version 2.0 (Applied Biosystems Technologies, Paisley, UK) on the basis of the consensus region of each of the sequences from the 10 genes of Lg8813 and Lg21881 strains (Table 1). cDNA was synthesized using the High Capacity RNA-to-cDNA Kit (Applied Biosystems). Amplification was performed in a 7900HT Fast Real-Time PCR Systems apparatus (Applied Biosystems) in triplicate for each sample using the FastStart Universal SYBR Green Master (Rox) (Roche Diagnostics, Basel, Switzerland) at Genomics Unit facilities (Parque Científico de Madrid). PCR amplification was initiated at 95°C for 10 min followed by 40 cycles at 95°C for 15 s and 60°C for 1 min. Fluorescence due to the binding of the SYBR Green to double-stranded DNA was measured at each cycle. The threshold cycle value (Ct) was obtained by automatic position of the threshold baseline at the mid-exponential phase of the curve. Data normalization and analysis were performed by means of RQ Manager Software Version 1.2. The 37°C condition was assigned as the reference sample for calculating relative gene expression. 

**Table 1:** Primers used for RT-qPCR experiments.

**Strain**	**Gene ID**	**Predicted protein function**	**Primer ID**	**Sequence 5’- 3’**
**Lg8831**	8831_c60_g79	Glycine betaine ABC transporter permease/substrate binding protein	g79c60_Forward	GCCCGGTTTCGTTTACTTGA
			g79c60_Reverse	AGGGACCACACCGATACCAA
	8831_c18_g15	Fructose operon transcriptional regulator	g15c18_Forward	TTTGCTTGGTGGTCGAGTGA
			g15c18_Reverse	GATGGCCGAACTGCCAAT
	8831_c49_g33	Cold-shock protein A	g33c49_Forward	CATCACTGCTGAAGATGGTACTGA
			g33c49_Reverse	GAAGCCATCGCTTTGAATTTG
	8831_c4_g16	Crp family transcriptional regulator	g16c4_Forward	AAAAGCGGAAGGGATATTAATTGA
			g16c4_Reverse	GAGATTCCACAAAATCCTGCTATGT
	8831_c96_g10	Glutamate decarboxylase	g10c96_Forward	CCGAAATCGAAAACCGTTGT
			g10c96_Reverse	AACTGTTCGTTTTCACTTGCATTC
**Lg21881**	21881_c16_g17	Pyridine mercuric reductase	g17c16_Forward	GGTGTTTTTGACCGTGATGAAG
			g17c16_Reverse	CTTCTGCCTGACCGTCTCTTG
	21881_c16_g19	TetR-family transcriptional regulator	g19c16_Forward	TCCGCTATTGCGCCAGAT
			g19c16_Reverse	CCCAGTTTAGCATTGCAAGCA
	21881_c63_g5	Glycerol uptake facilitator protein	g5c63_Forward	TCTGAACAAGACGAAAGCAACAG
			g5c63_Reverse	CAAAGCCCCAACCGAGTGT
	21881_c30_g6	Alcohol dehydrogenase	g6c30_Forward	GCAGGTAATGGTGCCGTCTT
			g6c30_Reverse	GCAGGTCTTCAACTGCACGAT
	21881_c50_g82	Activator of 2-hydroxyglutaryl-CoA dehydratase	g82c50_Forward	GGGCTGCGCGTTGTTGTTAAT
			g82c50_Reverse	CACTACTTGCGCTGCAGCAT
**Lg21881 & Lg8831**	*gyrA* *	DNA gyrase subunit A	gyrA_Forward	ACGGAATGAACGAGCTTGGTA
			gyrA_Reverse	CCGGTAATACGGGCAGATTTT

* Reference gene used in RT-qPCR experiments for both strains.

## Results and Discussion

### Global expression patterns of Lg8831 and Lg21881 in response to growth temperature

**Figure 1:**
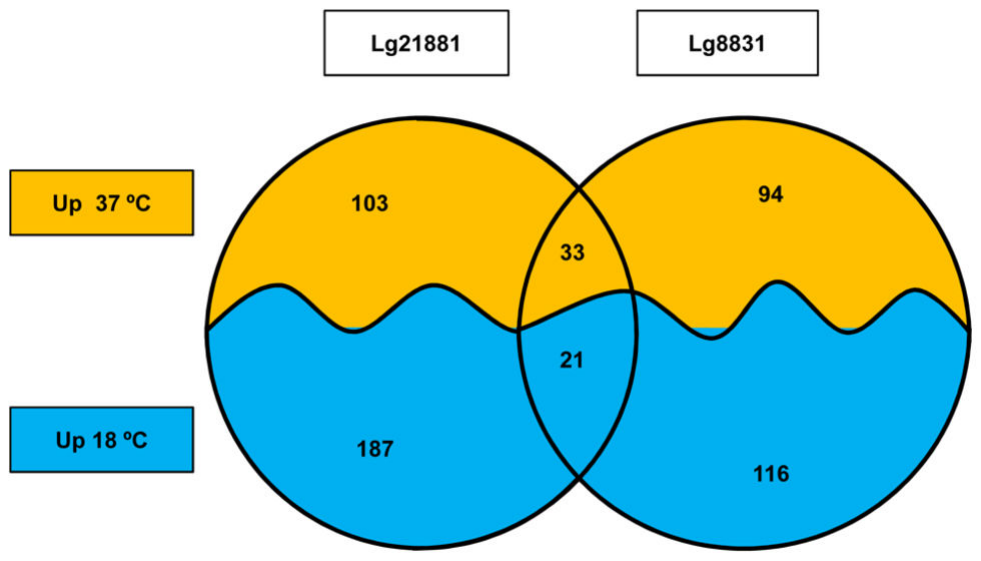
Comparison of the transcriptomic response of Lg8831 and Lg21881 grown at 18°C and 37°C. Up 37°C and Up 18°C represent up-regulated genes at each temperature.

We used whole-genome DNA microarrays to obtain a comprehensive overview of the molecular response of Lg8831 and Lg21881 strains grown at 18°C and 37°C. Differentially expressed genes detected with a 0.05% FDR and at least a two-fold change were selected. Under these conditions, 264 genes showed statistically significant differential expression for Lg8831: 137 (6.9% of its genome) and 127 genes (6.4% of its genome) were up-regulated at 18°C and 37°C, respectively (Figure 1). For Lg21881, 344 differentially expressed genes were identified: 208 (9.2% of its genome) up-regulated at 18°C and 136 (6.0% of its genome) at 37°C (Figure 1). These results are in accordance with other studies that observed that bacteria can modify the expression of approximately 10% of their genes in response to an increase or decrease in growth temperature [10,11,25]. Genes displaying a significant differential expression are listed in Tables S1-S4. 

The oligonucleotide array was designed to be optimized for the detection of each of the *L. garvieae* strains; therefore, for inter-strain comparisons, it was necessary to assess the similarity and specificity of the probes. Thus, this step allowed us to identify 1302 matching probes meeting the selection criteria used. Among these matching genes, we only found 21 genes up-regulated at 18°C in both strains (1.6% of the matching genes), and 33 genes up-regulated at 37°C in both strains (2.5% of the matching genes) (Figure 1). These results demonstrated that the core gene expression response to temperature is minimal, and evidenced a strain-specific variation in transcriptional responses to temperature of Lg8831 and Lg21881 strains.

**Figure 2:**
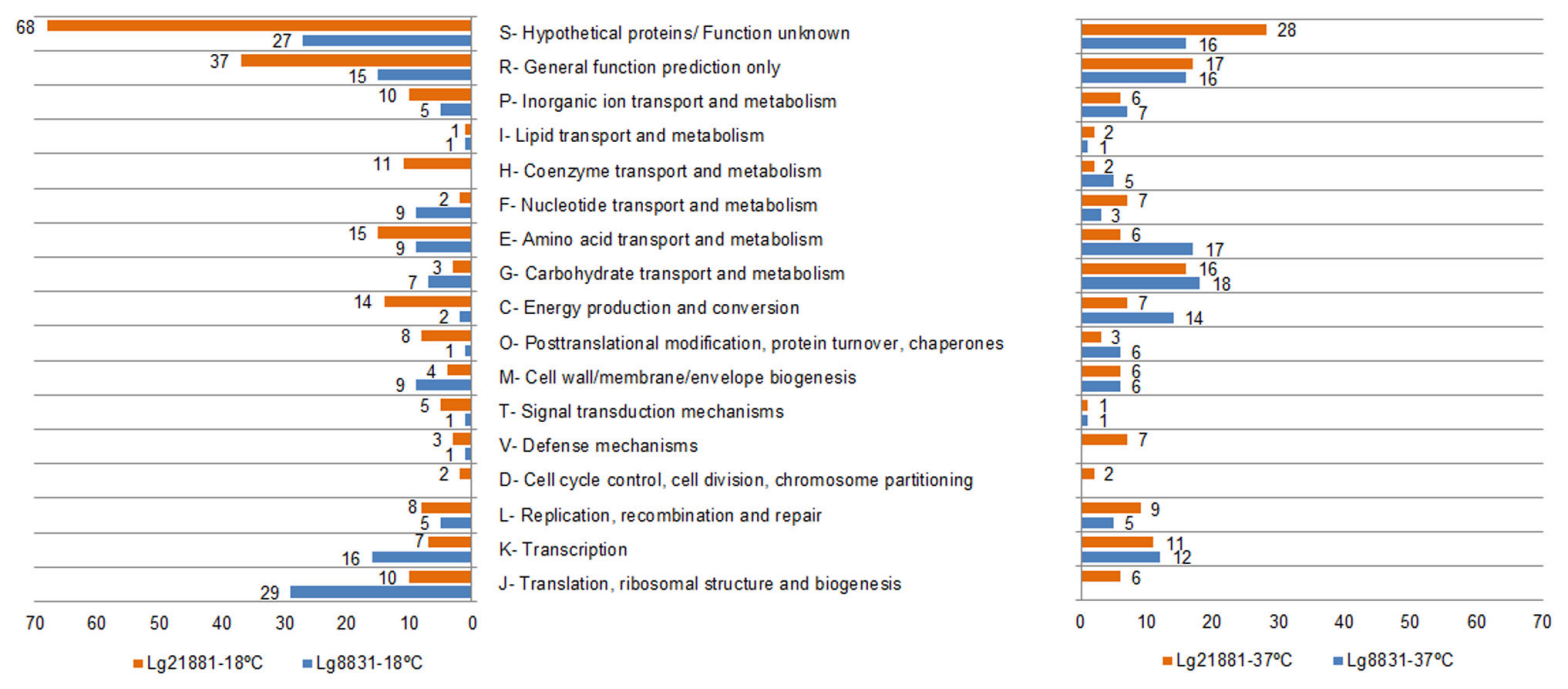
COG categories distribution of differentially expressed genes in Lg8831 and Lg21881 at 18°C and 37°C. Bars indicate the number of genes up-regulated in each functional group at each temperature in Lg8831 (blue) and Lg21881 (orange).

We categorized the differentially regulated genes based on the Clusters of Orthologous Groups (COG). As shown in Figure 2, the pattern and distribution of differentially expressed genes among COG categories was different for Lg8831 and Lg21881 strains and depended on the temperature.

In relation to the functional gene categories, Lg8831 and Lg21881 up-regulated at 37°C mainly genes within categories related to bacterial growth and metabolism such as COG categories G (carbohydrate transport metabolism), E (amino acid transport and metabolism) and K (transcription) (Figure 2). This result was expected considering that 37°C is the optimal growth temperature for this microorganism [7]. However, at 18°C the distribution pattern between gene categories varied greatly between strains (Figure 2). Thus, COG categories J (translation, ribosomal structure and biogenesis) and K (transcription) were the most represented at 18°C for Lg8831, whereas Lg21881 primarily up-regulated genes within the COG categories C (energy production and conversion) and H (coenzyme transport and metabolism) (Figure 2). Overall, these results (Figures 1 and 2) indicate a different transcriptional response of Lg8831 and Lg21881 to temperature, which was more evident at 18°C. The most significant differences observed in both strains are discussed in detail below. 

To assess the influence of the temperature in the growth kinetics of Lg8831 and Lg21881 and look for a possible relation between differences in their transcriptomes and differences in growth, we performed growth kinetics studies at 18°C and 37°C for both strains. At the same temperature (18°C or 37 °C), Lg8831 and Lg21881 exhibited similar growth rates (Table 2), but the growth rate at 37°C were higher for both strains compared to 18°C (p<0.05). However, at 18°C Lg8831 showed a shorter lag-phase (7.65 h) than that exhibited by Lg21881 (12.32 h) (Table 2) and this difference was statistically significant (p<0.05). The shorter lag-phase of Lg8831 grown at 18°C suggests that this strain could be better adapted to grow at low temperatures. 

**Table 2:** Growth kinetics parameters for Lg8831 and Lg21881 at 18°C and 37°C.

	**18°C**		**37°C**	
**Strain**	**Lg8831**	**Lg21881**	**Lg8831**	**Lg21881**
**Lag-phase (h)**	7.65 ± 0.60	12.32 ± 0.60	2.05 ± 0.10	2.1 ± 0.20
**Growth rate (h ^-1^ )**	0.14 ± 0.03	0.18 ± 0.05	0.63 ± 0.06	0.77 ± 0.05

### DNA microarray analysis verification

**Figure 3:**
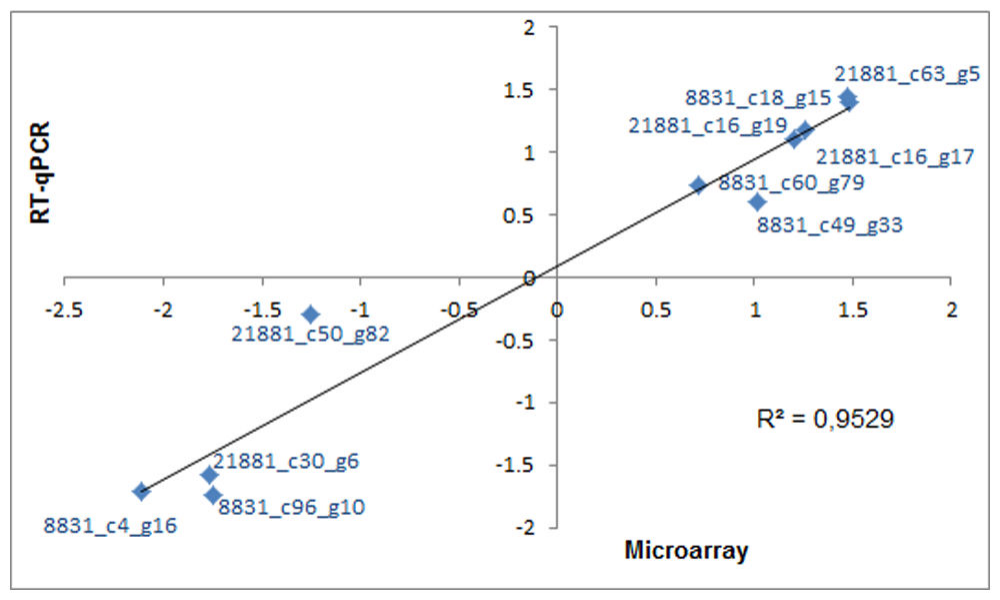
Correlation between RT-qPCR and microarray analysis data. The log_10_ values of the real-time RT-qPCR were plotted against the microarray analysis log_10_ values. The expression levels for 10 selected genes are shown.

To validate the microarray transcription profiling results, 10 differentially expressed genes were selected for quantitative RT-qPCR experiments with the same RNA samples used in the array hybridizations. Among them, six genes (three for each strain) were up-regulated at 18°C, and four genes (two for each strain) were repressed. There was a strong positive correlation (r=0.953) between the data obtained by both techniques (Figure 3). Thus, this strong correlation allowed us to validate the microarray results.

### Transcriptional profiling of Lg8831 and Lg21881 at 18°C compared to 37°C

#### Induction of a specific cold stress response in Lg8831

The functional analysis of the differentially up-regulated genes in Lg8831 at 18°C showed that a large number of genes were involved in translation, ribosomal structure and biogenesis (COG category J; 21%) and in transcription (COG category K; 12%). The up-regulation of genes within functional COG category J at 18°C could be implicated in the adaptation of Lg8831 to low temperature. Most of the up-regulated genes among this category corresponded to ribosomal proteins (Table S1). It has been previously shown that ribosomal proteins, translation factors and other components that help in the function and/or biogenesis of ribosomes are induced after exposure to low temperatures [26]. Ribosomal proteins act as chaperones ensuring accurate translation at low temperatures [27]. Furthermore, it is not surprising that a large proportion of genes involved in COG category K were up-regulated at 18°C, because is necessary the expression of specific transcription factors for the initiation of the transcription of cold-inducible genes whose products would assist in the adaptation to temperatures below the optimum [28]. 

**Figure 4:**
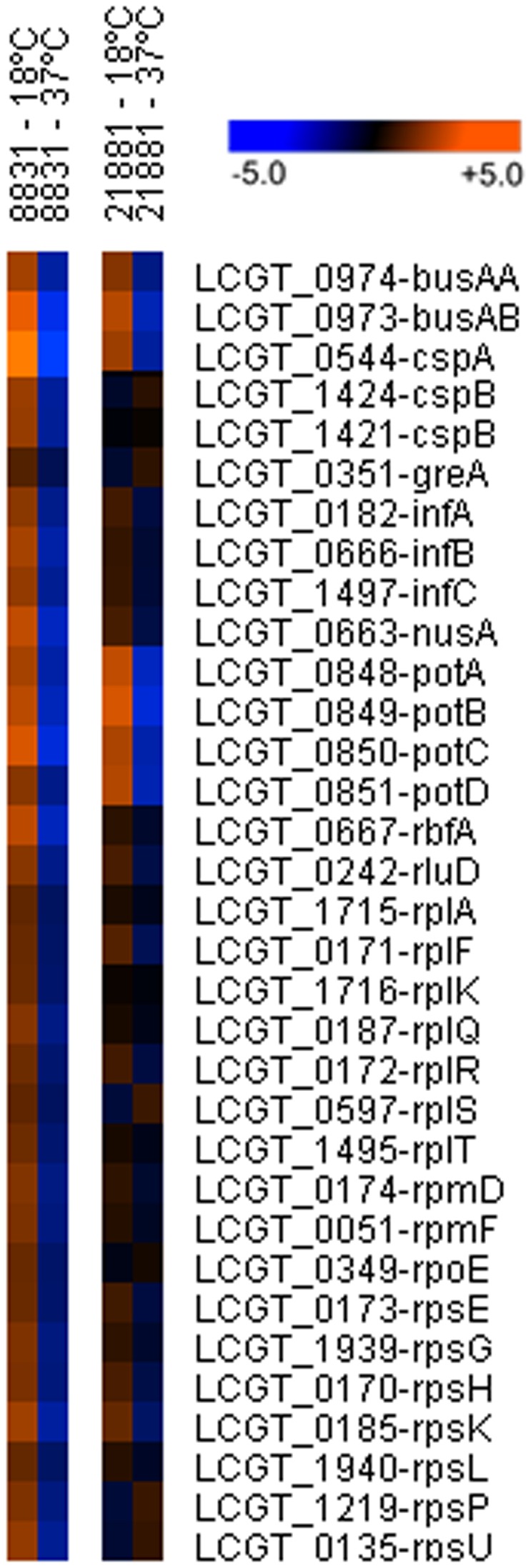
Comparison of the expression levels of cold stress-related genes in Lg8831 and Lg21881 at 18°C and 37°C by microarray analysis.

A great number of the genes among those found to be up-regulated at 18°C in Lg8831 encode proteins involved in a specific low-temperature response (Table S1, Figure 4). Thus, we observed the up-regulation of three *csp* homologues encoding cold-shock proteins (CSPs). The induction of CSPs has been described for a great number of bacteria exposed to low temperatures [26], mainly acting as RNA chaperones facilitating transcription and translation at low temperature in mesophilic bacteria [28,30]. We also observed the up-regulation of genes encoding previously described cold-induced proteins (CIPs), which are necessary for cellular adaptation to low temperature [26,31]. Some of these up-regulated genes include: genes involved in transcription such as *nusA* (transcription factor NusA) [30] and *greA* (transcription elongation factor GreA); and in translation, such as *infA* (translation initiation factor IF1) [32], *infB* (translation initiation factor IF2) [29], *infC* (translation initiation factor IF3) [33], and *rbfA* (ribosome binding factor A) [34,35] (Table S1). 

Transport systems allow bacteria to cope with environmental changes [31]. Genes encoding transport systems represented 23% of the up-regulated genes at 18°C, suggesting that the transport and secretion of molecules through the membrane could play significant role in the cold-adaptive process of Lg8831. Within this group, ABC transporters represented more than 50% (Table S1). Although energetically costly, ABC transporters usually have very high affinities for their solutes and catalyze transport at high rates [36]. Hence, induction of ABC transporters may allow Lg8831 to efficiently scavenge essential solutes giving it a competitive advantage when grown at low temperatures and assisting in cold adaptation. Transcription of *busAA* and *busAB* homologues encoding a glycine betaine uptake ABC transporter system, as well as transcription of the *potABCD* operon encoding the spermidine putrescine uptake ABC transport system, were found to be up-regulated at 18°C (Table S1). Both transport systems are important response mechanisms to cold conditions. Spermidine putrescine, and glycine betaine are compatible solutes that are known to accumulate during bacterial growth at low temperature, acting as cryoprotectants that prevent denaturing and the cold-induced aggregation of proteins [26,37,38]. 

Cryoprotectant substances are also believed to play a role in maintaining optimum membrane fluidity at low temperature [39]. Spermidine has been described as a bacteriolysis-inducing activator in staphylococci, bacilli, and in certain streptococcal species [40]; and pronounced cell wall breakdown has been associated to responses to stress conditions [41,42]. Interestingly, we found the up-regulation of three genes encoding autolytic cell-wall breaking enzymes (gene25_contig19, gene51_contig19 and gene20_contig36), which act as muramidases that hydrolyze the beta-1,4-linked polysaccharides of the peptidoglycan (Table S1). One of the regulation mechanisms of autolytic enzymes is the teichoic acids system [43,44]. In this sense, 4 genes encoding proteins implicated in teichoic acid biosynthesis were found to be up-regulated (*tagD, tagF, tagG, tagH*) (Table S1). Additionally, *epsL* (encoding exopolysaccharide biosynthesis protein) and *glmS* (encoding glucosamine-fructose-6-phosphate aminotransferase) homologues were also up-regulated. These two genes are related to biosynthetic processes of membrane components. Hence, genes related to membrane functions (COG category M) that represents 6.5% of the up-regulated genes at 18°C in Lg8831, appear to be linked to both biological processes: the degradation and the biosynthesis of membrane components. This observation could be related to the reorganization of membrane composition, which is associated with growth at low temperatures [26,28].

Another significant result was the pronounced up-regulation of homologues of fructose metabolism (*fru* operon: *fruRCA*) (Table S1). This operon is regulated by the repressor FruR in the absence of fructose in *Lactococcus lactis* and low-GC Gram-positive bacteria. The FruR effector is fructose-1-phosphate, which is necessary to relieve repression by FruR [45]. Therefore, the observed up-regulation of the *fru* operon in Lg8831 requires the presence of fructose-1-phosphate, which is likely generated as a consequence of the cell wall breakdown processes described above (KEGG pathway: lgr00520) that yields fructose-6-phosphate. This fructose-6-phosphate is then redirected to the glycolysis pathway and converted to fructose-1,6-biphosphate, which is further spontaneously dephosphorylated to fructose-1-phosphate [45]. 

Thus, a relevant finding of this work is that Lg8831 is able to sense and adapt to temperatures below its optimum by means of differential expression of genes whose products are involved in a specific cold response. Additionally, this specific and coordinated remodeling of the transcriptome could be related to the differential growth kinetics, associated with a shorter lag phase, demonstrated by this strain (Table 2).

#### Induction of a general stress response in Lg21881

The largest group among the up-regulated genes at 18°C (31%) was the phage, prophage, and chromosomal mobile elements. The up-regulation of this group of genes has been observed in several bacteria under different stress conditions [46-48] and phage-related proteins have been previously reported to be up-regulated in group A *Streptococcus* at 29°C compared to 37°C [8]. Phages, prophages, and mobile elements play a role in bacterial genome diversification, and there is evidence of the induction of genetic exchange mechanisms as a general response to stress in Gram-positive bacteria [49]. An explanation to this fact is that, at low growth rates, cells enter into a state favorable for DNA rearrangements [48].

Furthermore, we observed the up-regulation of some genes related to the cold-stress response that were also found to be up-regulated in Lg8831 at 18°C, such as *nusA*, *cspA* and the *potABCD* operon (Table S2, Figure 4). The Lg21881 *opuB* homologue was up-regulated at 18°C. The Opu transport system is described as involved in cryoprotection in bacteria, serving for the acquisition of a substantial number of osmoprotectants. It has been demonstrated that the Opu transporters mediate the uptake of the cryoprotectant solute glycine betaine in *Bacillus subtilis* cold-stressed cells [37]. In addition to *nusA*, *cspA*, *potABCD* and *opuB*, we also found up-regulated genes that have been widely described as general stress responsive genes (Table S2) such as: (i) two transcripts encoding OsmC (osmotically inducible protein C)-like proteins (gene32_contig62 and gene67_contig49), whose expression is regulated by multiple stress conditions [50]; (ii) the gene66_contig50 transcript, which encodes a general stress family protein; and (iii) the gene15_contig69, which encodes a Clp protease that has been described as general biomarker of stress adaptive behavior in a wide range of bacteria [51]. 

 Several genes encoding proteins presumed to be involved in iron homeostasis were up-regulated at 18°C in Lg21881. These genes include: the *fhu* operon (see below); genes of the iron ABC transport system (gene26_contig33, gene27_contig33, gene28_contig33 and gene29_contig33; see TS4); *sufB*, *sufC*, *sufD*, *sufS* homologues; and a gene encoding a NifU-like protein. Iron transporter proteins as well as other proteins involved in iron homeostasis are differentially expressed in response to growth temperatures in group A *Streptococcus* [8]. Suf and NifU proteins play a role in the iron-sulfur (Fe-S) cluster assembly machinery [52]. Bacterial Fe-S proteins are essential regulators of gene transcription under stress conditions, acting as sensors of the environment and enabling the organism to adapt to prevailing conditions [53]. These genes involved in iron homeostasis could assist Lg21881 to adapt to low temperature conditions contributing to the general stress response showed by this strain at 18°C.

We observed a strong induction of the *glpKDF* operon at 18°C (Table S2). Up-regulation of GlpD has also been reported in *Lactobacillus sakei* when exposed to low temperature [54]. It is known that glycerol metabolism is linked to membrane properties. Glycerol-3-phosphate can be converted to phosphatidic acid towards the activity of an acetyltransferase, which leads to membrane phospholipid synthesis that it is likely linked to changes in membrane composition associated to maintenance of membrane integrity when grown at low temperatures [28,54]. The observed up-regulation of an acetyltranferase transcript (gene8_contig37; see Table S3), which may be related to the synthesis of phosphatidic acid, makes sense in this light. Alternately, the activity of GlpD is also related to the induction of respiratory metabolism in Lg21881 when grown at 18°C (see below).

#### Aerobic respiration activation in Lg21881

Energy production and conversion, and coenzyme transport and metabolism (COG categories C and H) represented 13% of the up-regulated genes at 18°C in Lg21881. An important finding in this study is that 11 genes within these categories (45%) were related to an aerobic respiratory metabolism. LAB have usually been considered as fermentative bacteria, but in recent years some studies have noted the potential of the respiratory metabolism in different lactococcal strains, demonstrating that they undergo respiration and thrive when they are grown in the presence of oxygen and a heme source [55-57]. 

In *L. lactis* subsp. *lactis*, the membrane respiration chain requires dehydrogenases (membrane proteins NoxB and/or GlpD), menaquinones (encoded by *men* genes), and a terminal electron acceptor (the cytochrome oxidase encoded by *cyd* genes) [55,57]. At 18°C in Lg21881, we observed the up-regulation of homologues within the menaquinone biosynthesis pathway (*menB*, *menC*, *menD*, *menE*, *menH* and *ubiE*, see Table S2), and two homologues of the cytochrome oxidase synthesis operon (*cydC* and *cydD*). Additionally, the strong up-regulation of *glpD* (see above) is likely related to both independent biological processes occurring at 18°C in Lg21881: playing a role as a membrane dehydrogenase that is necessary for the aerobic respiration process, and in the biosynthesis of membrane phospholipids associated with membrane changes at low temperatures. 

Homologues within the *fhu* operon (*fhuB fhuC* and *fhuD*) encoding subunits of the iron complex ABC transport system were also up-regulated at 18°C. The *fhu* operon is responsible for heme uptake in respiring *L. lactis* cells [57] and is likely to be responsible for heme import from the external environment in Lg21881. Heme is required to activate a respiration metabolic pathway in streptococci such us *L. lactis* and *Streptococcus agalactiae* [55,58]. Because Lg21881 lacks the appropriate genes for heme biosynthesis, heme must be present in the medium to activate respiration. Although BHI broth was not supplemented with heme, trace amounts of heme in BHI medium have been reported to activate respiration-related genes [58]. 

We also observed in Lg21881 the up-regulation of genes encoding enzymes related to mixed-acid fermentation that could be associated to the respiratory metabolism such as proteins within the pyruvate dehydrogenase complex (*pdhABD* and dihydrolipoamide acetyltransferase homologues), *als* (acetolactate synthase), and *pycA* (pyruvate carboxylase) (Table S2). When oxygen and heme are present, *L. lactis* shifts to a mixed-acid fermentation, more complete glucose utilization, and energy generation by NADH oxidation via an electron transfer chain [36]. LAB lack a complete Krebs cycle, and consequently, NADH, which is required for the respiratory chain, is produced by carbon catabolism. Once phosphorylated, sugar is catabolized to pyruvate via glycolysis with production of ATP and NADH. Thus, pyruvate dehydrogenase (Pdh) provides extra NADH from pyruvate when oxygen is present [57]. Moreover, we observed in Lg21881 the up-regulation of NADH oxidase, an enzyme that in *L. lactis* is responsible for the shift to the mixed acid fermentation pathway in aerobically grown cells [59]. 

The up-regulation of respiration-related genes at 18°C in Lg21881 may be linked to the increased solubility of oxygen at lower temperatures, which may increase oxidative stress. Thus, the activation of respiration activity can protect cells against damage by consuming oxygen via respiratory metabolism [53]. We did not, however, observe a significant up-regulation of the respiratory pathways in strain Lg8831 at 18°C highlighting the possibility that this phenomenon is strain-dependent. The specific up-regulation of aerobic respiration-related genes in Lg21881 may be related to the general response to stress observed at 18°C for this strain. To the best of our knowledge, this is the first time respiratory metabolism has been described in *L. garvieae*.

### Transcriptional profiling of Lg8831 and Lg21881 at 37°C compared to 18°C

Carbohydrate transport- and metabolism-related genes (COG category G) were the largest group among the up-regulated genes in both strains (Figure 2), which is in accordance with the high growth rate and metabolic activity at 37°C shown by both strains (Table 2). 

We observed the up-regulation of genes involved in an elevated glycolytic activity at 37°C in Lg8831, which is likely related to the higher growth rate at 37°C compared to 18°C (Table 2). This group includes: (i) genes encoding glycolytic enzymes such as *gapB* (glyceraldehyde-3-phosphate dehydrogenase), *fbaA* (fructose-biphosphate aldolase), *scrK* (fructokinase) and *pyk* (pyruvate kinase); (ii) enzymes of fermentation pathways such as ldh (lactate dehydrogenase) and *pdhAB* (pyruvate dehydrogenase complex); and (iii) PTS systems, necessary for the transport and phosphorylation of sugars before their utilization and genes within the energy production and conversion pathways (Table S3). The growth of lactic acid bacteria is characterized by the generation of acidic end products of fermentation (mainly lactic acid), which accumulate in the extracellular milieu leading to an acidification of the environment [60]. In this regard, the expression of acid resistance-related genes could help to cope with these environmental changes. Thus, 17 of the up-regulated genes at 37°C in Lg8831 (13%) were related to an acid resistance response, including: (i) genes within the three main systems involved in lactococcal pH homeostasis: the F_1_F_0_-ATPase proton pump, the glutamate decarboxylase (GAD) system and the arginine deiminase (ADI) pathway [61]; (ii) the *lctO* gene encoding L-lactate oxidase, an enzyme that has been implicated in the removal of excess lactate that accumulates after fermentation [62]; and (iii) other described biomarkers of the acid resistance response such as *dnaK* and *groEL* [60], and *clpE* and *clpB* [51] (Table S3). 

In addition, 19 out of the 136 up-regulated transcripts at 37°C (14%) in Lg21881 corresponded to plasmid-encoded genes (Table S4). Lg21881 carries five circular plasmids encoding heterogeneous functions acquired by horizontal gene transfer from other lactic acid bacteria (LAB) [63]. The optimum growth temperature for *L. garvieae* is 37°C [7]. It is therefore expected that the expression of extra chromosomal material is favored at this temperature. 

### Responses potentially linked to the pathogenesis of *L. garvieae*

The influence of temperature in the expression of virulence factors has been extensively studied in different bacterial pathogens such as *Listeria monocytogenes* [64] and *Pseudomonas aeruginosa* [65]. In this study, several genes encoding proteins associated with the pathogenesis of different pathogens were up-regulated in Lg8831 at 18°C or in Lg21881 at 37°C.

 The *rpoE* homologue encoding the delta subunit of RNA polymerase, the cold-responsive *potABCD* operon, and three genes (gene25_contig19, gene51_contig19, gene20_contig36) encoding autolytic enzymes were up-regulated in Lg8831 at 18°C (Table S1). RpoE has been described as an essential global modulator of environmental adaptation in Gram-positive bacteria such us *S. mutans* [66], and it has also been linked to the virulence of *S. agalactiae* [67,68] and the fish pathogen *Vibrio harveyi* [69]. The cold-responsive *potABCD* operon has been described as necessary for the pathogenesis of *S. pneumoniae* in experimentally infected mice [70]. In particular, it has been demonstrated that PotD it is intricately linked to the fitness, survival, and pathogenesis of pneumococci in host microenvironments [71]. Autolytic enzymes also play an important role in the infectious process in Gram-positive pathogens [40]. When bacteriolysis occurs during infection, cell wall- and membrane-associated lipopolysaccharides (endotoxin), lipoteichoic acids, and peptidoglycan are released. These compounds can act on macrophages to induce the release of reactive oxygen and nitrogen species, cytotoxic cytokines, hydrolases, and proteinases, and also activate the coagulation and complement cascades. All these agents and processes are involved in the pathophysiology of septic shock and multiple organ failure resulting from severe microbial infection [40]. Lg8831 was isolated from rainbow trout suffering lactococcosis, a disease greatly influenced by temperature [1]. Therefore, the up-regulation of these genes in Lg8331 at 18°C could be relevant in the pathogenesis of lactococcosis, a disease characterized by a severe generalized septicemic process in affected fish. To confirm this hypothesis, our group is currently performing transcriptomic studies in experimentally infected trout. 

In Lg21881, genes that may be associated with pathogenesis were those related to manganese homeostasis up-regulated at 37°C: a *mtnR* homologue encoding a manganese-dependent transcriptional regulator of the DtxR family; *mtsABC* homologues encoding a manganese ABC transporter; and a *mntH* homologue encoding a Nramp family manganese transport protein (Table S4). MtnR was first described in *B. subtilis* as a transcriptional regulator, which responded to manganese. The manganese ABC transporter and MntH are the targets of MntR regulation [72]. Both, Nramp and ABC class manganese transporters have been extensively described as essential for the virulence of several Gram-positive pathogens [73]. In particular, MntH has been linked to the virulence of pathogens such as *Salmonella enterica* [74], *Escherichia coli* [75], *Brucella abortus* [76] and *Yersinia pestis* [77]. Thus, as in other pathogens, the regulation of the expression of these manganese transporters could also play a role in the pathogenesis of infections caused by Lg21881.

## Conclusion

The data coming from the whole genome DNA microarray of *L. garvieae* constructed in the present study demonstrated excellent agreement in the microarray validation experiments, indicating that it can be a useful tool for transcriptome studies in *L. garvieae*. In the present study, this microarray was used to perform the first transcriptome analysis of *L. garvieae*. This study of gene expression revealed that Lg8831 and Lg21881 differentially respond to temperature suggesting strain-specific adaptation mechanisms. In addition, it is the first time that a respiratory metabolism has been described in *L. garvieae*. These data extend our understanding of the regulatory networks and biology of this important pathogen.

## Supporting Information

Table S1 **Genes showing significant up-regulation by microarray hybridization in Lg8831 grown at 18°C compared to 37°C.**
(DOC)

Table S2 **Genes showing significant up-regulation by microarray hybridization in Lg21881 grown at 18°C compared to 37°C.**
(DOC)

Table S3 **Genes showing significant up-regulation by microarray hybridization in Lg8831 grown at 37°C compared to 18°C.**
(DOC)

Table S4 **Genes showing significant up-regulation by microarray hybridization in Lg21881 grown at 37°C compared to 18°C.**
(DOC)
