# Supplementary material for: Global Transcriptome Analysis of Lactococcus garvieae Strains in Response to Temperature
Source: PLoS One. 2013 Nov 4;8(11):e79692. doi: 10.1371/journal.pone.0079692 (PMC3817100; doi:10.1371/journal.pone.0079692)
Supplement: Table S1 — Genes showing significant up-regulation by microarray hybridization in Lg8831 grown at 18°C compared to 37°C. (DOC) [file pone.0079692.s001.doc]

**Table S1:** Genes showing significant up-regulation by microarray hybridization in Lg8831 grown at 18ºC compared to 37ºC.

| **COG category** | **Fold-change** | **Microarray gene ID** | **Predicted protein function** | ***L. garvieae***  **ATCC49156**  **gene ID** | **Gene**  **symbol** | **Group function** |
| --- | --- | --- | --- | --- | --- | --- |
| **Amino acid transport and metabolism** | 2.44 | TSno3_c036_g022 | Spermidine/putrescine ABC transporter substrate-binding protein | LCGT_0851 | potD | ABC transporters/Resp. to cold |
|  | 4.06 | TSno3_c036_g023 | Spermidine/putrescine ABC transporter permease component II | LCGT_0850 | potC | ABC transporters/Resp. to cold |
|  | 3.84 | TSno3_c036_g024 | Spermidine/putrescine ABC transporter permease component I | LCGT_0849 | potB | ABC transporters/Resp. to cold |
|  | 3.19 | TSno3_c036_g025 | Spermidine/putrescine ABC transporter ATP-binding protein | LCGT_0848 | potA | ABC transporters/Resp. to cold |
|  | 5.24 | TSno3_c060_g079 | Glycine betaine ABC transporter permease/substrate binding protein | LCGT_0973 | busAB | ABC transporters/Resp. to cold |
|  | 3.44 | TSno3_c060_g080 | Glycine betaine ABC transporter ATP binding protein | LCGT_0974 | busAA | ABC transporters/Resp. to cold |
|  | 2.26 | TSno3_c075_g005 | Amino acid transporter protein | LCGT_1943 |  | Transporters |
|  | 2.73 | TSno3_c078_g001 | Amine transmembrane transporter | LCGT_1695 |  | Transporters |
|  | 2.67 | TSno3_c012_g029 | Amino acid ABC transporter substrate-binding protein | LCGT_0723 |  | ABC transporters |
| **Carbohydrate transport and metabolism** | 12.13 | TSno3_c018_g013 | Fusion of IIA, IIB and IIC component of fructose-specific PTS | LCGT_0426 | fruA | PTS systems |
|  | 17.87 | TSno3_c018_g014 | 1-phosphofructokinase | LCGT_0425 | fruC | PTS systems |
|  | 2.08 | TSno3_c039_g001 | Transporter protein (MIP superfamily: Aquaporin/Glycerol uptake facilitator) | LCGT_0200 |  | Transporters |
|  | 2.43 | TSno3_c053_g009 | Exopolysaccharide biosynthesis protein | LCGT_1232 | epsL |  |
|  | 2.48 | TSno3_c060_g008 | Beta-glucosidase | LCGT_0933 |  |  |
|  | 2.67 | TSno3_c060_g104 | PTS system, glucose-specific IIA component | LCGT_0994 |  | PTS systems |
|  | 4.39 | TSno3_c075_g004 | Glucose uptake protein | LCGT_1944 | glcU |  |
| **Cell wall/membrane/envelope biogenesis** | 2.75 | TSno3_c035_g012 | Glycosyltransferase | LCGT_0890 |  |  |
|  | 2.21 | TSno3_c010_g034 | Teichoic acid transport system, ATP-binding protein | LCGT_1242 | tagH | ABC transporters |
|  | 2.33 | TSno3_c010_g035 | Teichoic acid transport system, ABC transporter permease protein | LCGT_1241 | tagG | ABC transporters |
|  | 3.4 | TSno3_c010_g037 | Glycerol-3-phosphate cytidyltransferase | LCGT_1236 | tagD |  |
|  | 2.83 | TSno3_c010_g039 | Teichoic acid biosynthesis protein F | LCGT_1234 | tagF |  |
|  | 3.26 | TSno3_c019_g025 | Lisozyme-like superfamily protein (hydrolysis of beta-1,4- linked polysaccharides) | LCGT_0295 |  | Autolysin |
|  | 2.48 | TSno3_c019_g051 | LytC lysozyme-like protein (autolysin) | LCGT_1278 |  | Autolysin |
|  | 3.86 | TSno3_c060_g153 | Glucosamine-fructose-6-phosphate aminotransferase | LCGT_1022 | glmS |  |
|  | 2.24 | TSno3_c089_g017 | Cell shape-determining protein MreC | LCGT_1911 | mreC |  |
| **Defense mechanisms** | 3.5 | TSno3_c058_g040 | Multidrug ABC transporter ATP-binding subunit | LCGT_0296 |  | ABC transporters |
| **Energy production and conversion** | 2.09 | TSno3_c010_g020 | Sodium/hydrogen antiporter | LCGT_1254 |  | Transporters |
|  | 2.46 | TSno3_c048_g059 | Dihydroorotate dehydrogenase electron transfer subunit | LCGT_1338 | pyrDII |  |
| **General function prediction only** | 4.58 | TSno3_c006_g018 | Basic membrane lipoprotein | LCGT_1841 | bmp |  |
|  | 2.12 | TSno3_c006_g025 | Major facilitator superfamily (MFS) transporter protein | LCGT_1846 |  | Transporters |
|  | 2.55 | TSno3_c013_g001 | Conserved hypothetical protein (rhodanese-related sulfurtransferase family protein) | LCGT_0762 |  |  |
|  | 2.08 | TSno3_c028_g010 | Universal stress protein | LCGT_1165 |  | Response to stress |
|  | 4.26 | TSno3_c036_g020 | Lisozyme-like superfamily protein (hydrolysis of beta-1,4- linked polysaccharides) | LCGT_0861 |  | Autolysin |
|  | 2.63 | TSno3_c036_g122 | ABC transporter ATP-binding protein | None |  | ABC transporters |
|  | 2.63 | TSno3_c037_g048 | Predicted RNA-binding protein (contains KH domain) | LCGT_1218 |  |  |
|  | 3.61 | TSno3_c038_g002 | ABC transporter ATP-binding protein | LCGT_0400 |  | ABC transporters |
|  | 2.05 | TSno3_c051_g007 | Predicted RNA-binding protein | LCGT_0070 |  |  |
|  | 6.08 | TSno3_c060_g013 | MFS-superfamily transporter protein | LCGT_0938 |  | Transporters |
|  | 2.1 | TSno3_c060_g122 | Transporter protein (similar to tsqA from *E.coli*) | LCGT_1006 |  | Transporters |
|  | 2.26 | TSno3_c068_g002 | ABC transporter ATP-binding protein/permease | LCGT_1444 |  | ABC transporters |
|  | 2.29 | TSno3_c077_g023 | Hypothetical protein (YceG-like superfamily) | LCGT_0350 |  |  |
|  | 2.29 | TSno3_c083_g011 | Hypothetical protein (RNA-binfing) | LCGT_1728 |  |  |
|  | 3.47 | TSno3_c029_g005 | Cell surface protein | LCGT_1035 |  |  |
| **Hypothetical proteins** | 2.05 | TSno3_c007_g030 | Hypothetical protein | None |  |  |
|  | 2.35 | TSno3_c010_g022 | Hypothetical protein | None |  |  |
|  | 3 | TSno3_c010_g038 | Hypothetical protein | LCGT_1235 |  |  |
|  | 2.18 | TSno3_c011_g001 | Hypothetical protein | None |  |  |
|  | 2.7 | TSno3_c012_g023 | Conserved hypothetical protein | LCGT_0729 |  |  |
|  | 2.48 | TSno3_c012_g048 | Hypothetical protein | LCGT_1091 |  |  |
|  | 2.06 | TSno3_c019_g014 | Hypothetical protein | None |  |  |
|  | 2.2 | TSno3_c019_g016 | Hypothetical protein | None |  |  |
|  | 2.86 | TSno3_c019_g023 | Hypothetical protein | None |  |  |
|  | 4.85 | TSno3_c019_g024 | Hypothetical protein | LCGT_1083 |  |  |
|  | 2.5 | TSno3_c019_g039 | Hypothetical protein | LCGT_1148 |  |  |
|  | 2.18 | TSno3_c019_g047 | Conserved hypothetical protein | LCGT_1275 |  |  |
|  | 2.56 | TSno3_c033_g003 | Hypothetical protein | LCGT_1402 |  |  |
|  | 2.34 | TSno3_c035_g011 | Hypothetical protein | LCGT_0889 |  |  |
|  | 3.11 | TSno3_c035_g018 | Hypothetical protein | None |  |  |
|  | 2.45 | TSno3_c036_g026 | Hypothetical protein | None |  |  |
|  | 2.45 | TSno3_c037_g034 | Hypothetical protein | LGCT_1204 |  |  |
|  | 5.48 | TSno3_c042_g028 | Hypothetical protein | LCGT_1760 |  |  |
|  | 3.84 | TSno3_c047_g030 | Hypothetical protein | LCGT_0665 |  |  |
|  | 4.58 | TSno3_c049_g054 | Hypothetical protein | LCGT_0531 |  |  |
|  | 4.66 | TSno3_c058_g039 | Hypothetical protein | None |  |  |
|  | 3.55 | TSno3_c060_g116 | Hypothetical protein | LCGT_0998 |  |  |
|  | 2.01 | TSno3_c060_g125 | Hypothetical protein (endonuclease-like) | None |  |  |
|  | 3.94 | TSno3_c060_g203 | Hypothetical membrane associated-protein | LCGT_1062 |  |  |
|  | 2.86 | TSno3_c068_g007 | Hypothetical protein | LCGT_1418 |  |  |
|  | 2.44 | TSno3_c096_g016 | Hypothetical protein | None |  |  |
|  | 2.17 | TSno3_c105_g002 | Hypothetical protein | None |  |  |
| **Inorganic ion transport and metabolism** | 2.38 | TSno3_c007_g019 | Zinc ABC transport system, ATP binding protein | LCGT_0180 | znuC | ABC transporters |
|  | 2.25 | TSno3_c007_g060 | Zinc ABC transport system, substrate binding protein | LCGT_0143 | znuA | ABC transporters |
|  | 2.32 | TSno3_c036_g019 | Cation-transporting E1-E2 type ATPase | LCGT_0862 |  | Transporters |
|  | 2.27 | TSno3_c060_g059 | Cation-transporting ATPase | LCGT_0959 |  | Transporters |
|  | 3.21 | TSno3_c066_g008 | Cation transmembrane efflux protein | LCGT_1078 |  | Transporters |
| **Lipid transport and metabolism** | 2.04 | TSno3_c049_g062 | Acetyl-CoA carboxylase carboxyl transferase subunit alpha | LCGT_0523 |  |  |
| **Nucleotide transport and metabolism** | 2.28 | TSno3_c010_g023 | Uracil transporter protein | LCGT_1252 |  | Transporters |
|  | 2.14 | TSno3_c048_g017 | Protein involved in ribonucleotide reduction | LCGT_1300 |  |  |
|  | 2.18 | TSno3_c048_g056 | Orotidine-5'-phosphate decarboxylase activity | LCGT_1336 | pyrF |  |
|  | 2.88 | TSno3_c048_g057 | Dihydroorotate dehydrogenase | LCGT_1337 | pyrD |  |
|  | 2.8 | TSno3_c048_g066 | Carbamoyl-phosphate synthase large subunit | LCGT_1343 | carB |  |
|  | 4.76 | TSno3_c060_g168 | Xanthine/uracil permease | LCGT_1036 | pbuX |  |
|  | 2.61 | TSno3_c060_g169 | Xanthine phosphoribosyltransferase | LCGT_1037 | xpt |  |
|  | 2.56 | TSno3_c077_g025 | Adenine phosphoribosyltransferase | LCGT_0348 | apt |  |
|  | 3.68 | TSno3_c085_g021 | Dihydroorotate dehydrogenase | LCGT_1508 | pyrDA |  |
| **Post translational modification, protein turnover, chaperones** | 2.12 | TSno3_c009_g024 | Peptide methionine sulfoxide reductase | LCGT_0494 | msrA |  |
| **Replication, recombination and repair** | 2.13 | TSno3_c014_g013 | Primosomal protein DnaI | LCGT_0636 | dnaI |  |
|  | 2.04 | TSno3_c014_g014 | Replication protein DnaB | LCGT_0635 | dnaB |  |
|  | 2 | TSno3_c023_g024 | Single-stranded DNA-binding protein | LCGT_1879 |  |  |
|  | 2.08 | TSno3_c049_g044 | Putative N6-adenine-specific DNA methylase | LCGT_0538 |  |  |
|  | 2.76 | TSno3_c077_g026 | Single strand DNA-specific exonuclease | LCGT_0347 | recJ |  |
| **Signal transduction mechanisms** | 2.38 | TSno3_c035_g013 | EAL superfamily protein | LCGT_0891 |  |  |
| **Transcription** | 2.09 | TSno3_c007_g014 | DNA-directed RNA polymerase subunit alpha | LCGT_0186 | rpoA |  |
|  | 2.58 | TSno3_c007_g018 | Zinc transport transcription regulator (transcriptional regulator of the zit/znu operon) | LCGT_0181 |  |  |
|  | 4.09 | TSno3_c008_g002 | Transcription elongation factor NusA | LCGT_0663 | nusA | Response to cold |
|  | 2.7 | TSno3_c014_g015 | Transcriptional repressor NrdR | LCGT_0634 | nrdR |  |
|  | 30.28 | TSno3_c018_g015 | Fructose operon transcriptional regulator | LCGT_0424 | fruR |  |
|  | 2.64 | TSno3_c035_g009 | Rgg/GadR/MutR family transcriptional activator | LCGT_0887 |  |  |
|  | 2.1 | TSno3_c053_g008 | Transcriptional regulator (LytR familiy) | LCGT_1231 |  |  |
|  | 2.75 | TSno3_c060_g140 | Transcriptional regulator | LCGT_1019 |  |  |
|  | 2.16 | TSno3_c065_g017 | DNA-directed RNA polymerase subunit omega | LCGT_1634 | rpoZ |  |
|  | 2.14 | TSno3_c077_g022 | Transcription elongation factor GreA | LCGT_0351 | greA | Response to cold |
|  | 2.13 | TSno3_c077_g024 | DNA-directed RNA polymerase subunit delta | LCGT_0349 | rpoE | Response to cold |
|  | 2.44 | TSno3_c078_g020 | Rgg/GadR/MutR family transcriptional activator | None |  |  |
|  | 3.07 | TSno3_c080_g025 | ATP-dependent RNA helicase | LCGT_0225 | rheA |  |
|  | 10.42 | TSno3_c049_g033 | Cold-shock protein A | LCGT_0544 | cspA | Response to cold |
|  | 2.8 | TSno3_c068_g010 | Cold shock protein B | LCGT_1421 | cspB | Response to cold |
|  | 2.73 | TSno3_c068_g011 | Cold shock protein B | LCGT_1424 | cspB | Response to cold |
| **Translation, ribosomal structure and biogenesis** | 2.44 | TSno3_c007_g013 | Ribosomal protein L17 | LCGT_0187 | rplQ | Ribosomal proteins/Resp. to cold |
|  | 2.58 | TSno3_c007_g015 | SSU ribosomal protein S11P | LCGT_0185 | rpsK | Ribosomal proteins/Resp. to cold |
|  | 2.28 | TSno3_c007_g017 | Translation initiation factor IF-1 | LCGT_0182 | infA | Response to cold |
|  | 2.66 | TSno3_c007_g027 | Large subunit ribosomal protein L30 | LCGT_0174 | rpmD | Ribosomal proteins/Resp. to cold |
|  | 2.11 | TSno3_c007_g028 | Small subunit ribosomal protein 5S | LCGT_0173 | rpsE | Ribosomal proteins/Resp. to cold |
|  | 2.14 | TSno3_c007_g029 | Large subunit ribosomal protein L18 | LCGT_0172 | rplR | Ribosomal proteins/Resp. to cold |
|  | 2.09 | TSno3_c007_g031 | 50S ribosomal protein L6 | LCGT_0171 | rplF | Ribosomal proteins/Resp. to cold |
|  | 2.27 | TSno3_c007_g033 | 30S ribosomal protein S8 | LCGT_0170 | rpsH | Ribosomal proteins/Resp. to cold |
|  | 2.89 | TSno3_c007_g067 | Small subunit ribosomal protein S21 | LCGT_0135 | rpsU | Ribosomal proteins/Resp. to cold |
|  | 3.74 | TSno3_c008_g003 | Ribosome maturation factor RimP | LCGT_0662 | rimP |  |
|  | 2.59 | TSno3_c014_g016 | tRNA (guanine-N7-)-methyltransferase | LCGT_0633 | trmB |  |
|  | 2.47 | TSno3_c017_g007 | Large ribosomal subunit, ribosomal protein L32 | LCGT_0051 | rpmF | Ribosomal proteins/Resp. to cold |
|  | 2.34 | TSno3_c037_g050 | 30S ribosomal protein S16 | LCGT_1219 | rpsP | Ribosomal proteins/Resp. to cold |
|  | 3.74 | TSno3_c047_g028 | Ribosome-binding factor A | LCGT_0667 | rbfA | Response to cold |
|  | 3.37 | TSno3_c047_g029 | Translation initiation factor IF-2 | LCGT_0666 | infB | Response to cold |
|  | 2 | TSno3_c048_g089 | 50S ribosomal protein L19 | LCGT_0597 | rplS | Ribosomal proteins/Resp. to cold |
|  | 2.24 | TSno3_c051_g010 | Ribonuclease P protein component | LCGT_0068 | rnpA |  |
|  | 2.4 | TSno3_c060_g166 | rRNA methylase | LCGT_1034 |  |  |
|  | 2.16 | TSno3_c065_g009 | rRNA methylase | LCGT_1628 |  |  |
|  | 2.08 | TSno3_c065_g010 | Methyonyl-tRNA formyltransferase | LCGT_1629 |  |  |
|  | 2.22 | TSno3_c075_g008 | 30S ribosomal protein S12 | LCGT_1940 | rpsL | Ribosomal proteins/Resp. to cold |
|  | 2.32 | TSno3_c075_g009 | 30S ribosomal protein S7 | LCGT_1939 | rpsG | Ribosomal proteins/Resp. to cold |
|  | 2.64 | TSno3_c077_g028 | Ribonuclease Z | LCGT_0345 | rnz |  |
|  | 2.03 | TSno3_c081_g027 | Ribosomal large subunit pseudouridine synthase | LCGT_0242 | rluD | Ribosomal proteins/Resp. to cold |
|  | 2.23 | TSno3_c083_g004 | 50S ribosomal protein L1 | LCGT_1715 | rplA | Ribosomal proteins/Resp. to cold |
|  | 2.24 | TSno3_c083_g005 | 50S ribosomal protein L11 | LCGT_1716 | rplK | Ribosomal proteins/Resp. to cold |
|  | 2 | TSno3_c083_g012 | Ribosome recycling factor | LCGT_1729 | frr |  |
|  | 2.14 | TSno3_c085_g008 | 50S ribosomal protein L20 | LCGT_1495 | rplT | Ribosomal proteins/Resp. to cold |
|  | 2.54 | TSno3_c085_g010 | Translation initiation factor IF-3 | LCGT_1497 | infC | Response to cold |
