# Supplementary material for: Global Transcriptome Analysis of Lactococcus garvieae Strains in Response to Temperature
Source: PLoS One. 2013 Nov 4;8(11):e79692. doi: 10.1371/journal.pone.0079692 (PMC3817100; doi:10.1371/journal.pone.0079692)
Supplement: Table S2 — Genes showing significant up-regulation by microarray hybridization in Lg21881 grown at 18°C compared to 37°C. (DOC) [file pone.0079692.s002.doc]

**Table S2:** Genes showing significant up-regulation by microarray hybridization in Lg21881 grown at 18ºC compared to 37ºC.

| **COG category** | **Fold-change** | **Microarray gene ID** | **Predicted protein function** | ***L. garvieae***  **ATCC49156 gene ID** | **Gene symbol** | **Group function** |
| --- | --- | --- | --- | --- | --- | --- |
| **Amino acid transport and metabolism** | 2.22 | HSno3_g16_c16 | Cystathionine beta-lyase | LCGT_0984 |  |  |
|  | 3.02 | HSno3_g105_c33 | Polar amino acid transport system ATP-binding protein | LCGT_0432 |  | ABC transporters |
|  | 3.34 | HSno3_g10_c37 | Serine hydroxymethyltransferase | LCGT_1527 | glyA |  |
|  | 2.11 | HSno3_g6_c39 | Amino acid ABC transporter ATP-binding protein | LCGT_0725 |  | ABC transporters |
|  | 3.43 | HSno3_g68_c49 | ABC amino acid transporter substrate-binding protein | LCGT_0912 |  | ABC transporters |
|  | 3.63 | HSno3_g5_c51 | Cysteine desulfurase / selenocysteine lyase | LCGT_0397 | sufS | Iron homeostasis |
|  | 2.93 | HSno3_g8_c62 | Spermidine/putrescine ABC_transporter substrate-binding protein | LCGT_0851 | potD | Response to cold |
|  | 2.96 | HSno3_g9_c62 | Spermidine/putrescine ABC transporter permease | LCGT_0850 | potC | Response to cold |
|  | 3.56 | HSno3_g11_c62 | Spermidine/putrescine ABC transporter permease | LCGT_0849 | potB | Response to cold |
|  | 3.08 | HSno3_g12_c62 | Spermidine/putrescine ABC transporter ATP-binding protein | LCGT_0848 | potA | Response to cold |
|  | 4.74 | HSno3_g9_c7 | Osmoprotectant transport system permease protein/glycine betaine/carnitine/choline ABC transporter permease and substrate-binding components | LCGT_1818 | opuB | Response to stress |
|  | 2.28 | HSno3_g28_c86 | Diaminopimelate decarboxylase | None | lysA |  |
|  | 4.23 | HSno3_g30_c86 | Dihydrodipicolinate reductase | None | dapB |  |
|  | 4.52 | HSno3_g35_c86 | N-acetyldiaminopimelate deacetylase | None |  |  |
|  | 4.45 | HSno3_g106_c33 | Amino acid ABC transporter substrate-binding component | LCGT_0431 |  | ABC transporters |
| **Carbohydrate transport and metabolism** | 2.43 | HSno3_g25_c16 | N-acetylglucosamine-specific PTS system IIABC components | LCGT_0994 |  | PTS system |
|  | 2.04 | HSno3_g31_c51 | Fructokinase | None | scrK |  |
|  | 29.39 | HSno3_g5_c63 | Glycerol uptake facilitator protein | LCGT_0790 | glpF | Response to cold |
| **Cell cycle control, cell division, chromosome partitioning** | 2.15 | HSno3_g1_c54 | Glucose inhibited division protein A (tRNA uridine 5-carboxymethylaminomethyl modification enzyme) | LCGT_1592 | gidA |  |
|  | 3.15 | HSno3_g34_c8 | Putative conjugative transfer protein: DNA_segregation ATPase, FtsK/SpoIIIE_family | None |  |  |
| **Cell wall/membrane/envelope biogenesis** | 2.05 | HSno3_g52_c16 | Glucosamine-fructose-6-phosphate aminotransferase | LCGT_1022 | glmS |  |
|  | 3.63 | HSno3_g25_c49 | Sortase A (surface protein transpeptidase) | LCGT_1291 | srtA |  |
|  | 2.18 | HSno3_g11_c86 | UDP-N-acetylglucosamine 2-epimerase | LCGT_1549 |  |  |
|  | 3.34 | HSno3_g60_c92 | Penicillin-binding protein 1A/1B | LCGT_0297 |  |  |
| **Coenzyme transport and metabolism** | 2.12 | HSno3_g56_c10 | Demethylmenaquinone methyltransferase (Menaquinone biosynthesis methyltransferase) | LCGT_1491 | ubiE | Aerobic respiration |
|  | 2.26 | HSno3_g32_c16 | Acetolactate synthase | LCGT_1004 | als | Miexd-acid fermentation |
|  | 2.03 | HSno3_g54_c16 | Folylpolyglutamate synthase | LCGT_1024 | folC |  |
|  | 3.21 | HSno3_g64_c2 | Thiamine biosynthesis lipoprotein | LCGT_0247 |  |  |
|  | 2.43 | HSno3_g30_c26 | 2-oxoglutarate decarboxylase | LCGT_0619 | menD | Aerobic respiration |
|  | 2.58 | HSno3_g31_c26 | 2-succinyl-6-hydroxy-2,4-cyclohexadiene-1-carboxylate synthase | LCGT_0618 | menH | Aerobic respiration |
|  | 2.12 | HSno3_g32_c26 | Naphthoate synthase | LCGT_0617 | menB | Aerobic respiration |
|  | 2.8 | HSno3_g33_c26 | O-succinylbenzoic acid-CoA ligase | LCGT_0616 | menE | Aerobic respiration |
|  | 2.74 | HSno3_g34_c26 | O-succinylbenzoate synthase | LCGT_0615 | menC | Aerobic respiration |
|  | 2.15 | HSno3_g32_c39 | Coproporphyrogen III oxidase | LCGT_1279 |  |  |
|  | 2.66 | HSno3_g6_c40 | Lipoate-protein ligase A | LCGT_0033 | lplA |  |
| **Defense mechanisms** | 2.45 | HSno3_g56_c2 | Multidrug transporter protein | LCGT_0253 |  |  |
|  | 3.65 | HSno3_g15_c39 | MFS family major facilitator transporter (tetracicline-resistance related) | LCGT_0716 |  |  |
|  | 2.72 | HSno3_g27_c61 | Multidrug transporter protein (macrolide efflux protein). Published. | LCGT_1286 | mdt(A) |  |
| **DNA replication, recombination, and repair** | 2.99 | HSno3_g17_c92 | Phage resolvase | LCGT_1131 |  | Phage-related proteins |
| **Energy production and conversion** | 3.59 | HSno3_g11_c10 | Cytochrome D ABC transporter ATP-binding/permase | LCGT_1446 | cydC | Aerobic respiration |
|  | 2.63 | HSno3_g12_c10 | Cytochrome D ABC transporter ATP-binding/permease | LCGT_1447 | cydD | Aerobic respiration |
|  | 18.03 | HSno3_g17_c16 | Pyridine mercuric reductase | LCGT_0985 |  |  |
|  | 2.56 | HSno3_g10_c40 | Pyruvate dehydrogenase complex, dihydrolipoamide dehydrogenase | LCGT_0029 | pdhD | Mixed-acid fermentation/ Aerobic respiration |
|  | 3.08 | HSno3_g7_c40 | Pyruvate dehydrogenase E1 component subunit alpha | LCGT_0032 | pdhA | Mixed-acid fermentation/ Aerobic respiration |
|  | 3.06 | HSno3_g8_c40 | Pyruvate dehydrogenase E1 component subunit beta | LCGT_0031 | pdhB | Mixed-acid fermentation/ Aerobic respiration |
|  | 2.99 | HSno3_g9_c40 | Pyruvate dehydrogenase component (dihydrolipoamide acetyltransferase) | LCGT_0030 | pdhC | Mixed-acid fermentation/ Aerobic respiration |
|  | 3.66 | HSno3_g35_c49 | Glutathione reductase | LCGT_0944 |  |  |
|  | 3.46 | HSno3_g4_c51 | NifU-like protein for Fe-S cluster formation | LCGT_0398 |  | Iron homeostasis |
|  | 16.81 | HSno3_g3_c63 | Glycerol kinase | LCGT_0788 | glpK | Response to cold |
|  | 19.52 | HSno3_g4_c63 | Glycerol-3-phosphate dehydorgenase | LCGT_0789 | glpD | Resp. to cold/ Aerobic respiration |
|  | 4.35 | HSno3_g9_c69 | Pyruvate carboxylase | LCGT_0360 | pycA | Mixed-acid fermentation |
|  | 2.69 | HSno3_g28_c79 | Na+/H+ antiporter | LCGT_1254 |  |  |
|  | 2.52 | HSno3_g46_c86 | L-lactate transporter protein | LCGT_1414 |  |  |
| **General function prediction only** | 2.36 | HSno3_g51_c10 | Hypothetical protein (glyoxalase family protein) | LCGT_1487 |  |  |
|  | 4.43 | HSno3_g55_c10 | Conserved hypothetical protein (glyoxalase/dioxygenase superfamily protein) | LCGT_1490 |  |  |
|  | 2.28 | HSno3_g3_c12 | Transporter protein | LCGT_0657 |  |  |
|  | 3.2 | HSno3_g42_c16 | Oxidoreductase | LCGT_1015 |  |  |
|  | 2.04 | HSno3_g32_c2 | ABC transporter substrate-binding protein | LCGT_0277 |  | ABC transporters |
|  | 2.82 | HSno3_g65_c2 | Hypothetical protein (Predicted flavoprotein) | LCGT_0246 |  |  |
|  | 2.56 | HSno3_g1_c26 | NADH oxidase | LCGT_0644 |  |  |
|  | 2.37 | HSno3_g35_c26 | Thioesterase superfamily protein | LCGT_0613 |  |  |
|  | 2.76 | HSno3_g36_c26 | Hypothetical protein (Predicted acetyltransferase) | LCGT_0612 |  |  |
|  | 2.64 | HSno3_g4_c26 | PTS system, ascorbate-specific IIC component | LCGT_0641 |  | PTS system |
|  | 3.11 | HSno3_g20_c27 | Hypothetical protein (predicted kinase) | LCGT_0744 |  |  |
|  | 2.7 | HSno3_g7_c27 | ABC transporter ATP binding protein | LCGT_0756 |  | ABC transporters |
|  | 3.91 | HSno3_g11_c37 | Lysozime-like superfamily protein | LCGT_1526 |  |  |
|  | 2.05 | HSno3_g8_c37 | Acetyltransferase | LCGT_1529 |  |  |
|  | 3.36 | HSno3_g18_c48 | HAD superfamily hydrolase | None |  |  |
|  | 3.83 | HSno3_g19_c48 | Cell wall anchor domain-containing protein | None |  |  |
|  | 3.25 | HSno3_g58_c48 | ABC transporter ATP binding protein/permease | LCGT_1755 |  | ABC transporters |
|  | 2.64 | HSno3_g59_c48 | ABC transporter ATP binding protein/permease | LCGT_1756 |  | ABC transporters |
|  | 2.63 | HSno3_g42_c49 | MFS family major facilitator transporter protein | LCGT_0938 |  |  |
|  | 2.01 | HSno3_g22_c50 | Flavin reductase family protein | LCGT_1166 |  |  |
|  | 2.15 | HSno3_g29_c50 | HAD superfamily hydrolase | LCGT_1172 |  |  |
|  | 3.64 | HSno3_g66_c50 | Gls24 family general stress protein | LCGT_1209 |  | Response to stress |
|  | 2.06 | HSno3_g67_c50 | Transporter protein (CorA family) | LCGT_1210 |  |  |
|  | 2.43 | HSno3_g28_c61 | Hypothetical protein (glyoxalase family) | LCGT_1285 |  |  |
|  | 3.63 | HSno3_g32_c62 | Conserved hypothetical protein (osmotically inducible protein C-like) | LCGT_0833 |  |  |
|  | 3.8 | HSno3_g34_c62 | (GATase1)-like domain containing protein | LCGT_0831 |  | Response to stress |
|  | 3.68 | HSno3_g1_c68 | Oxidoreductase | LCGT_0346 |  |  |
|  | 2.79 | HSno3_g16_c68 | Hypothetical protein (Metallo-beta-lactamase superfamily) | LCGT_0331 |  |  |
|  | 2.17 | HSno3_g4_c68 | NAD dependent epimerase/dehydratase family | LCGT_0343 |  |  |
|  | 3.9 | HSno3_g22_c69 | Oxidoreductase | LCGT_0346 |  |  |
|  | 3.3 | HSno3_g23_c8 | LPxTG-motif protein cell wall anchor domain protein | LCGT_1389 |  |  |
|  | 3.59 | HSno3_g29_c8 | XRE-family transcriptional regulator | None |  |  |
|  | 2.41 | HSno3_g2_c81 | Chloride channel protein | LCGT_0786 |  |  |
|  | 2.11 | HSno3_g4_c86 | ABC transporter ATP-binding protein | LCGT_1556 |  | ABC transporters |
|  | 2.09 | HSno3_g7_c86 | Glycosyltransferase | LCGT_1554 |  |  |
|  | 3.99 | HSno3_g20_c9 | MF superfamily transporter protein | LCGT_1846 |  |  |
|  | 2.53 | HSno3_g3_c86 | ABC transport system permease protein | LCGT_1557 |  | ABC transporters |
| **Hypothetical proteins** | 2.13 | HSno3_g52_c10 | Hypothetical protein | LCGT_1488 |  |  |
|  | 2.22 | HSno3_g6_c10 | Hypothetical protein | LCGT_0864 |  |  |
|  | 2.04 | HSno3_g100_c16 | Hypothetical protein | None |  |  |
|  | 2 | HSno3_g101_c16 | Hypothetical protein | None |  |  |
|  | 2.81 | HSno3_g108_c16 | Hypothetical protein | None |  |  |
|  | 17.95 | HSno3_g18_c16 | Conserved hypothetical protein | LCGT_0986 |  |  |
|  | 2.62 | HSno3_g91_c16 | Hypothetical protein | LCGT_1062 |  |  |
|  | 2.69 | HSno3_g94_c16 | Hypothetical protein | None |  |  |
|  | 3.7 | HSno3_g40_c26 | Hypotehtical protein | LCGT_1371 |  |  |
|  | 5.81 | HSno3_g19_c27 | Conserved hypothetical protein | LCGT_0745 |  |  |
|  | 3.54 | HSno3_g26_c27 | Hypothetical protein (Predicted membrane protein) | LCGT_0738 |  |  |
|  | 4.16 | HSno3_g11_c3 | Hypothetical phage protein | LCGT_1120 |  | Phage-related proteins |
|  | 5.36 | HSno3_g18_c3 | Hypothetical protein | LCGT_1129 |  | Phage-related proteins |
|  | 2.7 | HSno3_g43_c32 | Hypothetical protein | LCGT_0585 |  |  |
|  | 2.24 | HSno3_g3_c39 | Hypothetical protein | LCGT_0729 |  |  |
|  | 2.29 | HSno3_g9_c39 | Hypotehtical protein | LCGT_0722 |  |  |
|  | 2.62 | HSno3_g14_c41 | Hypothetical protein (pGL5_p41) | None |  | Plasmid |
|  | 2.2 | HSno3_g13_c46 | Hypothetical protein | LCGT_1648 |  |  |
|  | 2.52 | HSno3_g24_c49 | Hypothetical protein | None |  |  |
|  | 2.05 | HSno3_g5_c49 | Hypothetical protein | None |  |  |
|  | 3.02 | HSno3_g61_c50 | Hypothetical protein (Predicted membrane protein) | LCGT_1204 |  |  |
|  | 3.85 | HSno3_g63_c50 | Hypothetical protein | LCGT_1206 |  |  |
|  | 3.4 | HSno3_g64_c50 | Hypothetical protein | LCGT_1207 |  |  |
|  | 3.17 | HSno3_g65_c50 | Hypothetical protein | LCGT_1208 |  |  |
|  | 3.0 | HSno3_g2_c54 | Hypothetical protein | LCGT_1593 |  |  |
|  | 2.56 | HSno3_g35_c62 | Hypothetical protein (membrane protein) | LCGT_0830 |  |  |
|  | 2.46 | HSno3_g36_c62 | Hypothetical protein | LCGT_0829 |  |  |
|  | 5.68 | HSno3_g16_c66 | Hypothetical protein (phague-related) | LCGT_1792 |  | Phage-related proteins |
|  | 4.97 | HSno3_g17_c66 | Hypothetical phage protein | LCGT_1791 |  | Phage-related proteins |
|  | 2.51 | HSno3_g22_c66 | Hypothetical protein | None |  |  |
|  | 2.29 | HSno3_g26_c75 | Hypothetical protein | LCGT_1091 |  |  |
|  | 4.58 | HSno3_g13_c8 | Hypothetical protein | None |  |  |
|  | 3.29 | HSno3_g14_c8 | Hypothetical protein | None |  |  |
|  | 2.68 | HSno3_g17_c8 | Hyothetical protein | None |  |  |
|  | 3.81 | HSno3_g19_c8 | Hypothetical protein (transposon-related) | None |  |  |
|  | 3.97 | HSno3_g20_c8 | Hypothetical protein (transposon-related) | None |  |  |
|  | 3.39 | HSno3_g21_c8 | Hypothetical protein | None |  |  |
|  | 2.94 | HSno3_g22_c8 | Hypothetical protein | None |  |  |
|  | 3.66 | HSno3_g28_c8 | Hypothetical protein | None |  |  |
|  | 2.4 | HSno3_g35_c8 | Hypothetical protein | None |  |  |
|  | 4.83 | HSno3_g11_c84 | Hypothetical protein | None |  |  |
|  | 2.76 | HSno3_g8_c84 | Hypothetical protein | None |  |  |
|  | 2.54 | HSno3_g23_c86 | Hypothetical protein | LCGT_1539 |  |  |
|  | 2.97 | HSno3_g2_c9 | Hypothetical protein | None |  |  |
|  | 4.87 | HSno3_g15_c92 | Hypothetical protein | LCGT_1129 |  |  |
|  | 7.45 | HSno3_g21_c92 | Hypothetical protein (phague-related) | None |  | Phage-related proteins |
|  | 2.39 | HSno3_g25_c92 | Hypothetical protein (phague-related) | None |  | Phage-related proteins |
|  | 2.28 | HSno3_g34_c92 | Hypothetical protein (phague-related) | None |  | Phage-related proteins |
| **Inorganic ion transport and metabolism** | 2.31 | HSno3_g55_c2 | Sodium/hydrogen antiporter | LCGT_0254 |  |  |
|  | 3.85 | HSno3_g5_c28 | Sodium/hydrogen antiporter | LCGT_0893 |  |  |
|  | 3.59 | HSno3_g26_c33 | Iron ABC transporter substrate-binding protein | LCGT_0511 |  | Iron homeostasis |
|  | 4.12 | HSno3_g27_c33 | Iron ABC transporter, permease protein subunit B | LCGT_0510 |  | Iron homeostasis |
|  | 5.27 | HSno3_g28_c33 | Iron ABC transporter, permease protein subunit A | LCGT_0509 |  | Iron homeostasis |
|  | 4.07 | HSno3_g29_c33 | Iron ABC transporter, ATP-binding protein | LCGT_0508 |  | Iron homeostasis |
|  | 3.34 | HSno3_g8_c54 | Iron complex ABC transport system, ATP-binding protein | LCGT_1599 | fhuC | Aerobic respiration/Iron homeostasis |
|  | 3.41 | HSno3_g9_c54 | Iron complex ABC transport system, permease protein | LCGT_1600 | fhuB | Aerobic respiration/Iron homeostasis |
|  | 2.26 | HSno3_g10_c54 | Iron complex ABC transport system, substrate-binding protein | LCGT_1601 | fhuD | Aerobic respiration/Iron homeostasis |
|  | 3.06 | HSno3_g7_c64 | Cation efflux-family protein | LCGT_1078 |  |  |
| **Lipid metabolism** | 3.11 | HSno3_g61_c32 | Lipase | LCGT_0601 |  |  |
| **Nucleotide transport and metabolism** | 6.27 | HSno3_g42_c32 | Thioredoxin reductase | LCGT_0584 | trxB |  |
|  | 2.42 | HSno3_g10_c39 | Formate--tetrahydrofolate ligase | LCGT_0721 |  |  |
| **Post translational modification, protein turnover, chaperones** | 2.61 | HSno3_g11_c16 | NADH-peroxiredoxin reductase | LCGT_0979 |  |  |
|  | 2.97 | HSno3_g44_c33 | Peptide-methionine sulfoxide reductase | LCGT_0494 |  |  |
|  | 3.64 | HSno3_g67_c49 | Conserved hypothetical protein (osmotically inducible protein C-like) | LCGT_0913 |  | Response to stress |
|  | 3.15 | HSno3_g3_c51 | Predicted cysteine desulfurase activator complex subunit SufB | LCGT_0399 | sufB | Iron homeostasis |
|  | 3.13 | HSno3_g6_c51 | Fe-S cluster assembly protein SufD | LCGT_0396 | sufD | Iron homeostasis |
|  | 2.78 | HSno3_g7_c51 | SUF system: FeS cluster assembly protein ATP-dependent transporter | LCGT_0395 | sufC | Iron homeostasis |
|  | 2.5 | HSno3_g15_c69 | ATP-dependent Clp protease ATP-binding subunit | LCGT_0353 | clp | Response to stress |
|  | 2.36 | HSno3_g20_c75 | Gutathione peroxidase | LCGT_0866 |  |  |
| **Replication, recombination and repair** | 2.74 | HSno3_g2_c13 | DNA primase | LCGT_1591 | dnaG |  |
|  | 2.98 | HSno3_g3_c16 | DNA processing protein | LCGT_0971 |  |  |
|  | 3.15 | HSno3_g51_c16 | DNA repair protein RadC | LCGT_1021 | radC |  |
|  | 3.35 | HSno3_g21_c69 | Single-stranded-DNA-specific exonuclease | LCGT_0347 | recJ |  |
|  | 7.83 | HSno3_g3_c88 | Single-strand binding protein (phague-related) | LCGT_1136 |  | Phage-related proteins |
|  | 6.08 | HSno3_g7_c88 | Excisionase (phague-related) | None |  | Phage-related proteins |
| **Signal transduction mechanisms** | 2.08 | HSno3_g26_c16 | TspO/MBR family protein | LCGT_0995 |  |  |
|  | 2.32 | HSno3_g10_c28 | Diguanylate-cyclase family protein | LCGT_0888 |  |  |
|  | 2.54 | HSno3_g7_c28 | Hypothetical protein (EAL domain) | LCGT_0891 |  |  |
|  | 2.58 | HSno3_g107_c33 | Two-component sensor kinase | LCGT_0430 |  |  |
|  | 2.16 | HSno3_g8_c86 | EAL domain-containing protein | LCGT_1551 |  |  |
| **Transcription** | 15.9 | HSno3_g19_c16 | TetR-family transcriptional regulator | LCGT_0987 |  |  |
|  | 2.0 | HSno3_g11_c22 | Transcription termination factor NusA | LCGT_0663 | nusA | Response to cold |
|  | 3.26 | HSno3_g11_c28 | Rgg/GadR/MutR family transcriptional regulator | LCGT_0887 |  | Transcription factors |
|  | 2.36 | HSno3_g11_c32 | Cold-shock protein | LCGT_0544 | cspA | Response to cold |
|  | 3.26 | HSno3_g17_c48 | Transcriptional regulator | None |  |  |
|  | 2.05 | HSno3_g6_c82 | XRE-family transcriptional regulator | LCGT_1941 |  |  |
|  | 7.78 | HSno3_g19_c84 | XRE-family transcriptional regulator | None |  |  |
| **Translation, ribosomal structure ad biogenesis** | 4.99 | HSno3_g69_c49 | Amidase | LCGT_0911 |  |  |
|  | 2.33 | HSno3_g1_c16 | tRNA (uracil-5-)-methyltransferase | LCGT_0969 |  |  |
|  | 2.1 | HSno3_g129_c2 | 30S ribosomal protein S11 | LGCT_0185 | rpsK | Ribosomal proteins |
|  | 2.19 | HSno3_g158_c2 | 30S ribosomal protein S19 | LCGT_0157 | rpsS | Ribosomal proteins |
|  | 2.22 | HSno3_g9_c22 | Ribosomal protein L7Ae family protein | LCGT_0665 |  | Ribosomal proteins |
|  | 2.14 | HSno3_g9_c37 | Putative translation factor | LCGT_1528 |  |  |
|  | 2.2 | HSno3_g38_c54 | rRNA methylase | LCGT_1628 |  |  |
|  | 2.29 | HSno3_g39_c54 | Methyonyl-tRNA formyltransferase | LCGT_1629 |  |  |
|  | 3.79 | HSno3_g2_c68 | Ribonuclease Z | LCGT_0345 | rnz |  |
|  | 2.04 | HSno3_g37_c79 | tRNA (5-methylaminomethyl-2-thiouridylate)-methyltransferase | LCGT_1262 |  |  |
| **Other phage-related proteins** | 4.76 | HSno3_g1_c2 | Phague replication protein | None |  | Phage-related proteins |
|  | 3.07 | HSno3_g8_c2 | Phage antirepressor protein | LCGT_1143 |  | Phage-related proteins |
|  | 2.87 | HSno3_g9_c2 | Phage protein | None |  | Phage-related proteins |
|  | 2.34 | HSno3_g40_c29 | Phague infection protein | LCGT_0046 |  | Phage-related proteins |
|  | 3.27 | HSno3_g12_c3 | Phage protein | None |  | Phage-related proteins |
|  | 5.24 | HSno3_g14_c3 | Phage protein | None |  | Phage-related proteins |
|  | 2.02 | HSno3_g15_c3 | Phague protein | None |  | Phage-related proteins |
|  | 3.71 | HSno3_g19_c3 | Putative phage resolvase | LCGT_1131 |  | Phage-related proteins |
|  | 2.34 | HSno3_g5_c3 | Phague protein | LCGT_1117 |  | Phage-related proteins |
|  | 3.45 | HSno3_g19_c66 | Phage protein | LCGT_1790 |  | Phage-related proteins |
|  | 4.06 | HSno3_g26_c66 | Putative DNA-binding phage protein | None |  | Phage-related proteins |
|  | 3.03 | HSno3_g28_c66 | Phage related anti-repressor protein | None |  | Phage-related proteins |
|  | 3.89 | HSno3_g29_c66 | Putative DNA-binding phage protein | None |  | Phage-related proteins |
|  | 2.86 | HSno3_g10_c84 | Phage protein | None |  | Phage-related proteins |
|  | 4.51 | HSno3_g12_c84 | Putative phague protein | LCGT_1789 |  | Phage-related proteins |
|  | 3.57 | HSno3_g13_c84 | Phage protein (XRE-DNA binding protein) | None |  | Phage-related proteins |
|  | 5.04 | HSno3_g18_c84 | Phage related anti-repressor protein | None |  | Phage-related proteins |
|  | 3.68 | HSno3_g6_c84 | Phague protein | LCGT_1791 |  | Phage-related proteins |
|  | 2.25 | HSno3_g3_c92 | Phague protein | LCGT_1117 |  | Phage-related proteins |
|  | 3.31 | HSno3_g16_c92 | Phague protein | None |  | Phage-related proteins |
|  | 5.21 | HSno3_g32_c92 | Phage antirepressor protein | None |  | Phage-related proteins |
