# Supplementary material for: Global Transcriptome Analysis of Lactococcus garvieae Strains in Response to Temperature
Source: PLoS One. 2013 Nov 4;8(11):e79692. doi: 10.1371/journal.pone.0079692 (PMC3817100; doi:10.1371/journal.pone.0079692)
Supplement: Table S3 — Genes showing significant up-regulation by microarray hybridization in Lg8831 grown at 37°C compared to 18°C. (DOC) [file pone.0079692.s003.doc]

**Table S3:** Genes showing significant up-regulation by microarray hybridization in Lg8831 grown at 37ºC compared to 18ºC.

| **COG category** | **Fold-change** | **Microarray gene ID** | **Predicted protein function** | ***L. garvieae***  **ATCC49156 gene ID** | **Gene symbol** | **Group function** |
| --- | --- | --- | --- | --- | --- | --- |
| **Amino acid transport and metabolism** | **2.63** | TSno3_c005_g017 | Alanine dehydrogenase | LCGT_1812 | ald |  |
|  | **2.38** | TSno3_c005_g018 | Threonine dehydratase | LCGT_1811 |  |  |
|  | **3.85** | TSno3_c007_g107 | Arginine/ornithine antiporter | LCGT_0091 | arcD | Acid resistance |
|  | **4.76** | TSno3_c007_g108 | ornithine carbamoyltransferase | LCGT_0090 | arcB | Acid resistance |
|  | **5.56** | TSno3_c007_g109 | Arginine deiminase (Arginine dihydrolase) | LCGT_0089 | arcA | Acid resistance |
|  | **2.13** | TSno3_c010_g005 | Xaa-His dipeptidase | LCGT_0707 |  |  |
|  | **2.78** | TSno3_c023_g035 | L-glutamine synthetase | LCGT_1889 | glnA |  |
|  | **2.78** | TSno3_c037_g020 | Dipeptidase | LCGT_1190 | pepD |  |
|  | **2.44** | TSno3_c037_g022 | Xaa-His dipeptidase | LCGT_1192 |  |  |
|  | **4.00** | TSno3_c080_g015 | oligopeptide ABC transporter substrate-binding protein | LCGT_0216 | oppA | ABC transporters |
|  | **2.70** | TSno3_c080_g016 | Oligopeptide ABC transporter permease component | LCGT_0217 | oppB | ABC transporters |
|  | **27.03** | TSno3_c081_g024 | Lysine transporter protein | LCGT_0244 | lysQ |  |
|  | **4.55** | TSno3_c081_g035 | Amino acid permease | LCGT_0234 |  |  |
|  | **55.56** | TSno3_c096_g010 | Glutamate decarboxylase | None | gadB | Acid resistance |
|  | **45.45** | TSno3_c096_g011 | Glutamate/gamma-aminobutyrate antiporter | None | gadC | Acid resistance |
|  | **2.56** | TSno3_c080_g019 | Dipeptide/oligopeptide/nickel ABC trasporter ATP-binding subunit | LCGT_0220 | oppF | ABC transporters |
|  | **2.22** | TSno3_c042_g029 | Aminopeptidase | LCGT_1761 |  |  |
| **Carbohydrate transport and metabolism** | **2.04** | TSno3_c009_g033 | Mannose-specific PTS system IIAB components | LCGT_0487 |  | PTS system |
|  | **2.00** | TSno3_c009_g035 | Mannose-specific PTS system IID component | LCGT_0485 |  | PTS system |
|  | **2.13** | TSno3_c023_g006 | Glucose-6-phosphate isomerase | LCGT_1866 |  |  |
|  | **3.85** | TSno3_c024_g004 | PTS system sucrose-specific transporter subunit IIABC | LCGT_1646 | scrA | PTS system |
|  | **2.27** | TSno3_c028_g020 | PTS system beta-glucoside-specific transporter subunit IIABC | LCGT_1174 |  | PTS system |
|  | **2.27** | TSno3_c036_g005 | PTS system cellobiose-specific transporter subunit IIC | LCGT_0875 | celB | PTS system |
|  | **2.17** | TSno3_c048_g032 | Pyruvate kinase | LCGT_1315 | pyk | Glycolysis |
|  | **2.86** | TSno3_c051_g016 | Phosphocarrier protein (PTS HPr-like protein) | LCGT_0064 |  | PTS system |
|  | **2.33** | TSno3_c058_g032 | Phosphotransferase system cellobiose-specific component IIB | LCGT_0289 | celA | PTS system |
|  | **2.50** | TSno3_c060_g007 | Endoglycosidase (endo-beta-N-acetylglucosaminidase family) | LCGT_0932 |  |  |
|  | **2.50** | TSno3_c060_g113 | Chitinase (EndoS-like) | LCGT_0996 |  |  |
|  | **2.04** | TSno3_c065_g034 | Fructose-bisphosphate aldolase | LCGT_1652 | fbaA | Glycolysis |
|  | **2.00** | TSno3_c078_g005 | Galactokinase | LCGT_1698 |  | PTS system |
|  | **3.03** | TSno3_c085_g006 | Enolase | LCGT_1492 |  |  |
|  | **3.85** | TSno3_c089_g003 | glyceraldehyde-3-phosphate dehydrogenase | LCGT_1922 | gapB | Glycolysis |
|  | **2.33** | TSno3_c012_g039 | Phosphopentomutase | LCGT_0717 |  |  |
|  | **2.63** | TSno3_c024_g005 | Fructokinase | None | scrK | Glycolysis |
|  | **2.08** | TSno3_c028_g019 | Glycosidase (Predicted trehalose-6-phosphate hydrolase) | LCGT_1173 |  |  |
| **Cell wall/membrane/envelope biogenesis** | **2.17** | TSno3_c007_g035 | dTDP-L-rhamnose synthase | LCGT_0169 |  |  |
|  | **10.00** | TSno3_c048_g044 | Cyclopropane-fatty-acyl-phospholipid synthase | LCGT_1325 | cfa |  |
|  | **2.27** | TSno3_c049_g016 | Alanine racemase | LCGT_0561 | alr |  |
|  | **2.70** | TSno3_c018_g003 | Cell division protein ftsQ | LCGT_0435 | ftsQ |  |
|  | **2.38** | TSno3_c018_g004 | UDP-N-acetylglucosamine:LPS N-acetylglucosamine | LCGT_0434 | murG |  |
|  | **2.33** | TSno3_c018_g005 | UDP-N-acetylmuramoylalanine D-glutamate ligase | LCGT_0433 | murD |  |
| **Coenzyme transport and metabolism** | **2.04** | TSno3_c004_g021 | Phosphopantothenate--cysteine ligase | LCGT_1571 | coaB |  |
|  | **2.22** | TSno3_c023_g004 | Phosphopantetheine adenylyltransferase | LCGT_1864 |  |  |
|  | **2.27** | TSno3_c049_g017 | Holo-acyl carrier protein synthase | LCGT_0560 | acpS |  |
|  | **2.50** | TSno3_c060_g016 | Pyruvate oxidase | LCGT_0941 | pox |  |
|  | **2.27** | TSno3_c077_g014 | Dephospho-CoA kinase | LCGT_0356 | coaE |  |
| **DNA replication, recombination, and repair** | **2.08** | TSno3_c006_g011 | Predicted DNA alkylation repair enzyme | LCGT_1835 |  |  |
| **Energy production and conversion** | **3.23** | TSno3_c007_g004 | Alcohol dehydrogenase/oxidoreductase activity. | LCGT_0194 |  |  |
|  | **3.23** | TSno3_c007_g036 | L-lactate dehydrogenase | LCGT_0168 | ldh | Glycolysis/ Fermenation |
|  | **3.33** | TSno3_c032_g007 | Pyruvate-flavodoxin oxidoreductase | LCGT_0303 | nifJ |  |
|  | **2.27** | TSno3_c041_g007 | ATP synthase F0F1 subunit epsilon | LCGT_0413 | atpC | Acid resistance |
|  | **3.33** | TSno3_c041_g008 | ATP synthase F0F1 subunit beta | LCGT_0412 | atpD | Acid resistance |
|  | **3.23** | TSno3_c041_g009 | ATP synthase F0F1 subunit gamma | LCGT_0411 | atpG | Acid resistance |
|  | **2.63** | TSno3_c041_g010 | F0F1 ATP synthase, alpha subunit | LCGT_0410 | atpA | Acid resistance |
|  | **2.78** | TSno3_c041_g011 | F0F1 ATP synthase, delta subunit | LCGT_0409 | atpH | Acid resistance |
|  | **2.94** | TSno3_c041_g012 | F0F1 ATP synthase subunit B | LCGT_0408 | atpF | Acid resistance |
|  | **3.13** | TSno3_c041_g014 | ATP synthase F0F1 subunit C | LCGT_0406 | atpE | Acid resistance |
|  | **2.44** | TSno3_c043_g003 | Pyruvate dehydrogenase E2 component (dihydrolipoamide acetyltransferase) | LCGT_0030 |  |  |
|  | **2.27** | TSno3_c043_g004 | Pyruvate dehydrogenase E1 component beta subunit | LCGT_0031 | pdhB | Glycolysis/ Fermentation |
|  | **2.63** | TSno3_c043_g005 | Pyruvate dehydrogenase E1 component subunit alpha | LCGT_0032 | pdhA | Glycolysis/ Fermentation |
|  | **5.56** | TSno3_c060_g015 | L-lactate oxidase | LCGT_0940 | lctO | Acid resistance |
| **General function prediction only** | **111.11** | TSno3_c004_g015 | Transporter protein (MFS family) | LCGT_1577 |  |  |
|  | **2.78** | TSno3_c005_g003 | SAM-dependent methyltransferases | LCGT_1822 |  |  |
|  | **2.63** | TSno3_c008_g004 | LrgA family protein | LCGT_0661 |  |  |
|  | **2.13** | TSno3_c008_g005 | LrgB family protein | LCGT_0660 |  |  |
|  | **2.04** | TSno3_c009_g066 | Predicted HD superfamily hydrolase | LCGT_0458 |  |  |
|  | **2.13** | TSno3_c009_g077 | ABC transporter ATP-binding protein | LCGT_0447 |  | ABC transporters |
|  | **3.57** | TSno3_c013_g018 | HAD-like superfamily hydrolase | LCGT_0747 |  |  |
|  | **2.38** | TSno3_c018_g016 | ArsC family protein | LCGT_0423 |  |  |
|  | **2.27** | TSno3_c023_g003 | PDZ domain-containing protein | LCGT_1863 |  |  |
|  | **2.50** | TSno3_c023_g019 | Regulatory protein RecX | LCGT_1873 | recX |  |
|  | **2.78** | TSno3_c036_g032 | Conserved hypothetical protein (PTS IIB domain) | LCGT_0841 |  | PTS system |
|  | **2.78** | TSno3_c042_g016 | Putative proton-coupled thiamine transporter | LCGT_1749 |  |  |
|  | **2.94** | TSno3_c049_g057 | Short-chain type dehydrogenase | LCGT_0528 |  |  |
|  | **3.13** | TSno3_c060_g019 | Putative acetyltransferase | LCGT_0943 |  |  |
|  | **2.38** | TSno3_c077_g013 | Multidrug transporter protein (MFS superfamily) | LCGT_0357 |  |  |
|  | **2.78** | TSno3_c060_g115 | Chitin binding protein | LCGT_0997 |  |  |
| **Hypothetical proteins** | **2.86** | TSno3_c060_g006 | Conserved hypothetical protein | LCGT_0931 |  |  |
|  | **2.70** | TSno3_c004_g003 | Hypothetical protein | None |  |  |
|  | **38.46** | TSno3_c004_g014 | Hypothetical protein | LCGT_1578 |  |  |
|  | **2.17** | TSno3_c012_g037 | Hypothetical protein | LCGT_0718 |  |  |
|  | **2.33** | TSno3_c023_g005 | Hypothetical protein (predicted metyltransferse) | LCGT_1865 |  |  |
|  | **2.13** | TSno3_c023_g021 | Hypothetical protein | LCGT_1875 |  |  |
|  | **3.33** | TSno3_c035_g043 | Hypothetical protein | LCGT_0908 |  |  |
|  | **3.45** | TSno3_c036_g106 | Conserved hypothetical protein | LCGT_0787 |  |  |
|  | **2.22** | TSno3_c042_g013 | Hypothetical protein | LCGT_1747 |  |  |
|  | **2.13** | TSno3_c042_g030 | Hypothetical protein | LCGT_1762 |  |  |
|  | **2.13** | TSno3_c042_g031 | Hypothetical protein | LCGT_1763 |  |  |
|  | **2.17** | TSno3_c042_g060 | Conserved hypothetical protein | LCGT_1803 |  |  |
|  | **4.00** | TSno3_c049_g028 | Hypothetical protein | None |  |  |
|  | **2.44** | TSno3_c060_g017 | Hypothetical protein | LCGT_0942 |  |  |
|  | **2.33** | TSno3_c081_g032 | Hypothetical protein | LCGT_0237 |  |  |
|  | **2.13** | TSno3_c090_g002 | Hypothetical protein | LCGT_1373 |  |  |
| **Inorganic ion transport and metabolism** | **2.08** | TSno3_c007_g094 | Cation-transporting ATPase | LCGT_0104 |  |  |
|  | **3.45** | TSno3_c017_g006 | Heavy metal-(Cd/Co/Hg/Pb/Zn)-translocating P-type ATPase | LCGT_0052 |  |  |
|  | **2.94** | TSno3_c049_g029 | Heavy metal-(Cd/Co/Hg/Pb/Zn)-translocating P-type ATPase | LCGT_0548 |  |  |
|  | **11.76** | TSno3_c060_g001 | NRAMP-family manganese transport protein MntH | LCGT_0926 | mntH |  |
|  | **3.45** | TSno3_c085_g022 | Manganese ABC transporter ATP-binding protein | LCGT_1509 |  | ABC transporters |
|  | **3.03** | TSno3_c085_g023 | Manganese ABC transporter permease component | LCGT_1510 |  | ABC transporters |
|  | **2.56** | TSno3_c085_g024 | Manganese ABC transporter substrate-binding component | LCGT_1511 | mtsA | ABC transporters |
| **Lipid metabolism** | **12.50** | TSno3_c053_g004 | Activator of 2-hydroxyglutaryl-CoA dehydratase | LCGT_1227 | yxcA |  |
| **Nucleotide transport and metabolism** | **3.13** | TSno3_c026_g007 | Nucleoside diphosphate kinase | LCGT_0920 |  |  |
|  | **3.33** | TSno3_c028_g017 | Phosphoribosylamine-glycine ligase | LCGT_1171 | purD |  |
|  | **2.17** | TSno3_c042_g065 | Anaerobic ribonucleoside-triphosphate reductase | LCGT_1806 | nrdD |  |
| **Post translational modification, protein turnover, chaperones** | **2.63** | TSno3_c004_g004 | ATP-dependent Clp protease ATP-binding subunit ClpE | LCGT_1589 | clpE | Acid resistance |
|  | **2.78** | TSno3_c006_g013 | Trypsin-like serine protease, DegP/HtrA family | LCGT_1837 |  |  |
|  | **2.86** | TSno3_c018_g017 | Molecular chaperone DnaK | LCGT_0422 | dnaK | Acid resistance |
|  | **4.55** | TSno3_c026_g009 | Chaperone ClpB protein | LCGT_0919 | clpB | Acid resistance |
|  | **2.33** | TSno3_c042_g061 | Anaerobic ribonucleoside-triphosphate reductase activating protein | LCGT_1804 | nrdG |  |
|  | **2.78** | TSno3_c058_g009 | Chaperonin GroEL | LCGT_0268 | groEL | Acid resistance |
| **Replication, recombination and repair** | **3.70** | TSno3_c002_g001 | DNA repair protein recO (Recombination protein O), involved in DNA repair and recF pathway recombination | LCGT_0028 | recO |  |
|  | **2.17** | TSno3_c017_g005 | Chromosome segregation helicase | LCGT_0053 |  |  |
|  | **2.56** | TSno3_c081_g001 | Methylated-DNA-[protein]-cysteine S-methyltransferase | LCGT_0259 |  |  |
|  | **2.50** | TSno3_c081_g002 | Methylated-DNA-[protein]-cysteine S-methyltransferase | LCGT_0258 |  |  |
| **Signal transduction mechanisms** | **14.71** | TSno3_c081_g022 | Amino acid ABC transporter substrate-binding subunit | LCGT_0245 |  | ABC transporters |
| **Transcription** | **129.87** | TSno3_c004_g016 | Crp family transcription regulator | LCGT_1576 |  |  |
|  | **2.22** | TSno3_c010_g006 | GntR family transcriptional regulator | LCGT_0708 |  |  |
|  | **2.70** | TSno3_c013_g019 | Transcriptional regulator | LCGT_0746 |  |  |
|  | **3.70** | TSno3_c015_g002 | MerR superfamily transcriptional regulator | LCGT_0045 |  |  |
|  | **2.13** | TSno3_c023_g020 | Transcriptional regulator | LCGT_1874 |  |  |
|  | **2.04** | TSno3_c032_g011 | Transcription regulator | LCGT_0300 |  |  |
|  | **4.00** | TSno3_c048_g030 | Rgg/GadR/MutR-family transcription regulator | LCGT_1313 |  |  |
|  | **3.45** | TSno3_c049_g030 | Transcription regulator (Ars-like) | LCGT_0547 |  |  |
|  | **2.08** | TSno3_c049_g081 | Transcription regulator (LacI-GalR family repressors) | LCGT_0503 |  |  |
|  | **2.86** | TSno3_c053_g005 | tetR-family transcriptional regulator | LCGT_1228 | tetR |  |
|  | **10.00** | TSno3_c060_g002 | Transcription repressor protein (Mn-dependent transcriptional regulator) | LCGT_0927 |  |  |
|  | **2.50** | TSno3_c060_g014 | Transcriptional regulator (Spx family) | LCGT_0939 |  |  |
