# Supplementary material for: Global Transcriptome Analysis of Lactococcus garvieae Strains in Response to Temperature
Source: PLoS One. 2013 Nov 4;8(11):e79692. doi: 10.1371/journal.pone.0079692 (PMC3817100; doi:10.1371/journal.pone.0079692)
Supplement: Table S4 — Genes showing significant up-regulation by microarray hybridization in Lg21881 grown at 37°C compared to 18°C. (DOC) [file pone.0079692.s004.doc]

**Table S4:** Genes showing significant up-regulation by microarray hybridization in Lg21881 grown at 37ºC compared to 18ºC.

| **COG category** | **Fold-change** | **Microarray gene ID** | **Predicted protein function** | ***L. garvieae***  **ATCC49156 gene ID** | **Gene symbol** | **Group functions** |
| --- | --- | --- | --- | --- | --- | --- |
| **Amino acid transport and metabolism** | 3.33 | HSno3_g77_c2 | Aminoacid permease | LCGT_0234 |  |  |
|  | 3.33 | HSno3_g20_c32 | Metallo-dipeptidase | LCGT_0556 | pepV |  |
|  | 2.86 | HSno3_g8_c49 | Aspartate-ammonia ligase (Asparagine synthetase A) | LCGT_0961 | asnA |  |
|  | 2.94 | HSno3_g50_c50 | Dipeptidase D | LCGT_1192 | pepD |  |
|  | 7.14 | HSno3_g16_c7 | Threonine_dehydratase | LCGT_1811 |  |  |
|  | 8.33 | HSno3_g15_c7 | Alanine dehydrogenase | LCGT_1812 |  |  |
| **Carbohydrate transport and metabolism** | 2.00 | HSno3_g16_c13 | Phosphoglucomutase | LCGT_1574 |  |  |
|  | 2.63 | HSno3_g6_c15 | N-acetylglucosamine-6-phosphate deacetylase | LCGT_1317 | nagA |  |
|  | 5.26 | HSno3_g27_c16 | Chitinase | LCGT_0996 | chiA |  |
|  | 2.13 | HSno3_g18_c2 | 6-phospho-beta-glucosidase | LCGT_0291 | bglA |  |
|  | 2.17 | HSno3_g15_c2 | PTS system cellobiose transporter subunit IIA | LCGT_0294 | celC | PTS systems |
|  | 2.38 | HSno3_g16_c2 | PTS system cellobiose transporter subunit IIB | LCGT_0293 | celA | PTS systems |
|  | 2.50 | HSno3_g20_c2 | PTS system cellobiose transporter subunit IIB | LCGT_0289 | celA | PTS systems |
|  | 2.63 | HSno3_g19_c2 | PTS system, cellobiose-specific IIC component | LCGT_0290 | celB | PTS systems |
|  | 2.00 | HSno3_g53_c33 | Mannose-specific PTS system IID component | LCGT_0485 |  | PTS systems |
|  | 2.33 | HSno3_g52_c33 | Mannose-specific PTS system IIC component | LCGT_0486 |  | PTS systems |
|  | 2.08 | HSno3_g4_c4 | Glyceraldehyde 3-phosphate dehydrogenase | LCGT_1922 | gapA |  |
|  | 2.44 | HSno3_g31_c49 | Putative ribose transporter protein | LCGT_0948 |  |  |
|  | 2.94 | HSno3_g32_c49 | D-ribose pyranase (ABC-type ribose transport system, auxiliary component) | LCGT_0947 | rbsD |  |
|  | 3.70 | HSno3_g33_c49 | Ribokinase | LCGT_0946 | rbsK |  |
|  | 2.44 | HSno3_g3_c79 | Glucosamine-6-phosphate isomerase | LCGT_1230 |  |  |
|  | 4.35 | HSno3_g31_c79 | 6-phosphogluconate dehydrogenase | LCGT_1257 |  |  |
| **Cell cycle control, cell division, chromosome partitioning** | 2.22 | HSno3_g102_c33 | Cell division protein FtsQ | LCGT_0435 |  |  |
|  | 2.13 | HSno3_g7_c52 | Cell division protein DivIC (FtsB) | LCGT_0010 |  |  |
| **Cell wall/membrane/envelope biogenesis** | 4.55 | HSno3_g11_c31 | Choloylglycine hydrolase (CGH)-like protein | LCGT_0048 |  |  |
|  | 2.00 | HSno3_g40_c32 | Alanine-adding enzyme MurN | LCGT_0582 | murN |  |
|  | 2.56 | HSno3_g48_c32 | Serine/alanine-adding enzyme | LCGT_0589 | murM |  |
|  | 2.08 | HSno3_g103_c33 | UDP-N-acetylglucosamine:LPS N-acetylglucosamine transferase | LCGT_0434 | murG |  |
|  | 2.33 | HSno3_g104_c33 | UDP-N-acetylmuramoylalanine D-glutamate ligase | LCGT_0433 | murD |  |
|  | 2.00 | HSno3_g5_c82 | D-alanyl-D-alanine carboxypeptidase (penicillin-binding protein) | LCGT_1942 |  |  |
| **Coenzime transport and metabolism** | 2.33 | HSno3_g8_c30 | Phosphopantetheine adenylyltransferase | LCGT_1864 | coaD |  |
|  | 4.55 | HSno3_g4_c53 | 2-dehydropantoate 2-reductase (orf7_pGL3) | None | panE | Plasmid-encoded proteins |
| **Defense mechanisms** | 3.03 | HSno3_g6_c101 | Bacteriocin (pGL1_p6) | None |  | Plasmid-encoded proteins |
|  | 2.08 | HSno3_g6_c102 | Bacteriocin immunity-like protein (pGL2_p4) | None |  | Plasmid-encoded proteins |
|  | 2.27 | HSno3_g7_c102 | Putative bacteriocin (pGL2_p3) |  |  | Plasmid-encoded proteins |
|  | 4.55 | HSno3_g6_c41 | Bacteriocin ABC transporter permease (pGL5_lgnD) | None | lgnD | Plasmid-encoded proteins |
|  | 5.88 | HSno3_g5_c41 | Bacteriocin ABC transporter ATP-binding protein (pGL5_lgnC) | None | lgnC | Plasmid-encoded proteins |
|  | 7.14 | HSno3_g3_c41 | Putative bacteriocin (pGL5_p51) | None |  | Plasmid-encoded proteins |
|  | 7.69 | HSno3_g4_c41 | Bacteriocin immunty protein (pGL5_lgnI) | None |  | Plasmid-encoded proteins |
| **DNA replication, recombination, and repair** | 2.13 | HSno3_g11_c20 | Integrase/recombinase pGL4 | None |  | Plasmid-encoded proteins |
|  | 2.04 | HSno3_g22_c26 | Type I restriction-modification system, restriction subunit | None |  |  |
|  | 2.44 | HSno3_g17_c41 | Plasmid replication protein repB (pGL5) | LCGT_0627 | repB | Plasmid-encoded proteins |
|  | 2.44 | HSno3_g16_c41 | Plasmid segregation protein parA (pGL5) | LCGT_1230 | parA | Plasmid-encoded proteins |
|  | 2.22 | HSno3_g48_c42 | Protein involved in initiation of plasmid replication RepA (pGL5) | None | repA | Plasmid-encoded proteins |
|  | 4.00 | HSno3_g49_c42 | Protein involved in initiation of plasmid replication RepB (pGL5) | None | repB | Plasmid-encoded proteins |
|  | 2.04 | HSno3_g2_c49 | Site-specific tyrosine recombinase XerC | LCGT_0967 |  |  |
|  | 2.50 | HSno3_g8_c9 | Predicted DNA alkylation repair enzyme | LCGT_1835 |  |  |
| **Energy production and conversion** | 8.33 | HSno3_g47_c26 | Fe-S oxidoreductase | LCGT_1425 |  |  |
|  | 9.09 | HSno3_g48_c26 | Fe-S cluster binding protein | LCGT_1426 |  |  |
|  | 58.82 | HSno3_g6_c30 | Alcohol dehydrogenase/acetaldehyde dehydrogenase | LCGT_1862 | adhE |  |
|  | 15.63 | HSno3_g68_c50 | Fumarate reductase flavoprotein subunit | LCGT_1212 | frdA |  |
|  | 4.55 | HSno3_g13_c69 | Pyruvate-formate lyase | LCGT_0355 | pfl |  |
|  | 2.63 | HSno3_g2_c79 | Glycerol_kinase | LCGT_1229 | glpK |  |
|  | 3.33 | HSno3_g44_c92 | Pyruvate-flavodoxin oxidoreductase | LCGT_0303 | nifJ |  |
| **General function prediction only** | 2.94 | HSno3_g120_c2 | Acetyltransferase | LCGT_0192 |  |  |
|  | 3.23 | HSno3_g1_c22 | Linoleate isomerase | LCGT_0673 | lai |  |
|  | 2.22 | HSno3_g52_c26 | GNAT family acetyltransferase | None |  |  |
|  | 2.78 | HSno3_g37_c29 | Major facilitator family transporter protein | LCGT_0049 |  |  |
|  | 2.04 | HSno3_g9_c30 | Hypothetical protein (methyltransferase) | LCGT_1865 |  |  |
|  | 2.08 | HSno3_g18_c30 | Hypothetical protein (glyoxalase family protein ) | None |  |  |
|  | 2.17 | HSno3_g81_c33 | Hypothetical protein (HD superfamily hydrolase) | LCGT_0458 |  |  |
|  | 2.27 | HSno3_g18_c4 | CHAP domain protein (putative surface antigen) | LCGT_1908 |  |  |
|  | 3.13 | HSno3_g9_c49 | Acetyltransferase | LCGT_0960 |  |  |
|  | 2.13 | HSno3_g2_c5 | Hypothetical membrane protein | LCGT_1928 |  |  |
|  | 2.56 | HSno3_g81_c50 | Oxidoreductase | LCGT_1226 |  |  |
|  | 3.85 | HSno3_g12_c50 | ABC-type transport system, periplasmic component/surface lipoprotein (BmpA-like) | LCGT_1156 |  |  |
|  | 5.56 | HSno3_g5_c53 | PTS system, IIC component family protein (orf8_pGL3) | None |  | Plasmid-encoded proteins |
|  | 2.38 | HSno3_g3_c7 | Hypothetical protein (methyltransferase) | LCGT_1822 |  |  |
|  | 2.94 | HSno3_g14_c75 | Hypothetical protein (Predicted acetyltransferase) | LCGT_0872 |  |  |
|  | 2.94 | HSno3_g10_c82 | OPT-family protein (oligopeptide transporter protein) | LCGT_1937 |  |  |
|  | 4.76 | HSno3_g28_c16 | Chitin-binding protein | LCGT_0997 |  |  |
| **Hypothetical proteins** | 2.56 | HSno3_g12_c2 | Hypothetical protein | None |  |  |
|  | 2.63 | HSno3_g73_c2 | Conserved hypothetical protein | LCGT_0238 |  |  |
|  | 2.70 | HSno3_g13_c2 | Hypothetical protein | None |  |  |
|  | 2.17 | HSno3_g69_c2 | Hypothetical protein | None |  |  |
|  | 4.55 | HSno3_g5_c2 | Conserved hypothetical protein | None |  |  |
|  | 2.08 | HSno3_g16_c20 | Hypothetical protein (p14_pGL4) | None |  | Plasmid-encoded proteins |
|  | 2.44 | HSno3_g18_c26 | Hypothetical protein (HAD superfamily) | LCGT_0627 |  |  |
|  | 5.26 | HSno3_g49_c26 | Conserved hypothetical_protein | LCGT_1427 |  |  |
|  | 2.00 | HSno3_g21_c29 | Hypothetical protein | LCGT_0066 |  |  |
|  | 2.38 | HSno3_g4_c3 | Hypothetical protein | None |  |  |
|  | 3.13 | HSno3_g3_c4 | Hypothetical protein | LCGT_1923 |  |  |
|  | 2.04 | HSno3_g41_c42 | Hypothetical proteins (pGL5p34) | LCGT_1865 |  | Plasmid-encoded proteins |
|  | 2.08 | HSno3_g40_c42 | Hypothetical protein (pGL5p33) | LCGT_0434 |  | Plasmid-encoded proteins |
|  | 2.33 | HSno3_g4_c42 | Hypothetical protein (pGL5p03) | None |  | Plasmid-encoded proteins |
|  | 2.33 | HSno3_g34_c46 | Conserved hypothetical protein | LCGT_1672 |  |  |
|  | 11.24 | HSno3_g80_c49 | Hypothetical protein | LCGT_0903 |  |  |
|  | 2.04 | HSno3_g4_c5 | Hypothetical protein | None |  |  |
|  | 2.17 | HSno3_g6_c53 | Transposase (pseudo) (pGL3) | None |  | Plasmid-encoded proteins |
|  | 2.86 | HSno3_g13_c63 | Hypothetical protein | LCGT_0797 |  |  |
|  | 2.00 | HSno3_g6_c66 | Hypothetical protein | LCGT_1803 |  |  |
|  | 3.85 | HSno3_g27_c66 | Hypothetical protein | None |  |  |
|  | 2.04 | HSno3_g9_c75 | Hypothetical protein | LCGT_0877 |  |  |
|  | 2.08 | HSno3_g10_c75 | Hypothetical protein | LCGT_0876 |  |  |
|  | 3.03 | HSno3_g11_c82 | Hypothetical protein (PqqD-domain) | LCGT_1936 |  |  |
|  | 2.22 | HSno3_g9_c9 | Hypotehtical protein | LCGT_1836 |  |  |
|  | 2.38 | HSno3_g64_c92 | Hypotehtical protein | None |  |  |
|  | 3.13 | HSno3_g63_c92 | Hypothetical protein | None |  |  |
|  | 14.49 | HSno3_g45_c92 | Hypothetical protein | LCGT_0302 |  |  |
| **Inorganic ion transport and metabolism** | 2.63 | HSno3_g24_c16 | Cooper exporting ATPase | LCGT_0993 | copA |  |
|  | 3.33 | HSno3_g35_c29 | Heavy metal-(Cd/Co/Hg/Pb/Zn)-translocating P-type ATPase | LCGT_0052 |  |  |
|  | 6.25 | HSno3_g54_c49 | NRAMP family manganese transport protein | LCGT_0926 | mntH | Manganese homeostais |
|  | 2.78 | HSno3_g3_c85 | Manganese/iron ABC transport system ATPase component | LCGT_1509 | mtsC | Manganese homeostais/ ABC transporters |
|  | 4.55 | HSno3_g2_c85 | Manganese/iron ABC transport system permease protein | LCGT_1510 | mtsB | Manganese homeostais / ABC transporters |
|  | 7.69 | HSno3_g1_c85 | Manganese/iron ABC transporter substrate-binding lipoprotein | LCGT_1511 | mtsA | Manganese homeostais / ABC transporters |
| **Lipid transport and metabolism** | 17.86 | HSno3_g82_c50 | Activator of (R)-2-hydroxyglutaryl-CoA dehydratase | LCGT_1227 |  |  |
|  | 4.55 | HSno3_g12_c74 | Long-chain acyl-CoA synthetase | LCGT_0697 | fadD |  |
| **Nucleotide transport and metabolism** | 2.38 | HSno3_g37_c46 | Adenylosuccinate synthetase | LCGT_1679 | purA |  |
|  | 3.03 | HSno3_g60_c49 | Nucleoside diphosphate kinase | LCGT_0920 | ndk |  |
|  | 3.03 | HSno3_g10_c66 | Ribonucleoside-triphosphate reductase | LCGT_1806 | nrdD |  |
|  | 2.17 | HSno3_g5_c75 | dCMP deaminase | LCGT_0881 |  |  |
|  | 2.94 | HSno3_g1_c75 | IMP dehydrogenase/GMP reductase | LCGT_0885 | guaC |  |
|  | 5.00 | HSno3_g17_c75 | Orotate phosphoribosyltransferase | LCGT_0869 | pyrE |  |
|  | 5.56 | HSno3_g27_c79 | Uracil phosphoribosyltransferase/pyrimidine operon regulatory protein | LCGT_1253 | pyrR |  |
| **Post translational modification, protein turnover, chaperones** | 3.33 | HSno3_g68_c2 | Peptidyl-prolyl cis-trans isomerase A (cyclophilin A) | LCGT_0243 | ppiA |  |
|  | 2.13 | HSno3_g64_c33 | tmRNA-binding protein SmpB | LCGT_0474 | smpB |  |
|  | 2.04 | HSno3_g10_c9 | Protease | LCGT_1837 |  |  |
| **Replication, recombination and repair** | 2.86 | HSno3_g3_c31 | Resolvase | None |  |  |
| **Signal transduction mechanisms** | 2.04 | HSno3_g7_c30 | Hypothetical protein (PDZ domanin containing-protein) | LCGT_1863 |  |  |
| **Transcription** | 2.63 | HSno3_g11_c2 | XRE-family transcriptional regulator | LCGT_0312 |  |  |
|  | 2.70 | HSno3_g10_c29 | Transcriptional pleiotropic repressor | LCGT_0077 | codY |  |
|  | 2.04 | HSno3_g10_c32 | Catabolite control protein A | LCGT_0543 |  |  |
|  | 2.27 | HSno3_g34_c33 | Maltose operon-like transcription regulator | LCGT_0503 |  |  |
|  | 2.22 | HSno3_g17_c39 | Transcriptional regulator | LCGT_0715 |  |  |
|  | 2.17 | HSno3_g61_c48 | Transcriptional regulator | LCGT_1758 |  |  |
|  | 3.70 | HSno3_g34_c49 | Ribose operon repressor | LCGT_0945 | rbsR |  |
|  | 8.33 | HSno3_g53_c49 | DtxR family transcriptional repressor, Mn-dependent transcriptional regulator | LCGT_0927 | mtnR | Manganese homeostais |
|  | 2.33 | HSno3_g12_c68 | Regulatory protein Spx | LCGT_0335 |  |  |
|  | 2.94 | HSno3_g35_c68 | Phage transcription repressor | LCGT_0312 |  |  |
|  | 3.23 | HSno3_g1_c79 | tetR_family transcriptional_regulator | LCGT_1228 |  |  |
| **Translation, ribosomal structure and biogenesis** | 2.78 | HSno3_g33_c15 | 23S rRNA pseudouridine2457 synthase | LCGT_1342 | rluE |  |
|  | 2.17 | HSno3_g11_c30 | Valyl-tRNA synthetase | LCGT_1867 | valS |  |
|  | 2.44 | HSno3_g48_c30 | Arginyl-tRNA synthetase | LCGT_1900 | argS |  |
|  | 3.85 | HSno3_g56_c33 | Seryl-tRNA synthetase | LCGT_0482 | serS |  |
|  | 3.57 | HSno3_g15_c46 | Threonyl-tRNA synthetase | LCGT_1653 | thrS |  |
|  | 2.44 | HSno3_g30_c49 | Putative ribosome biogenesis GTPase RsgA 1 | LCGT_0949 |  |  |
